# Core set construction and association analysis of *Pinus massoniana* from Guangdong province in southern China using SLAF-seq

**DOI:** 10.1038/s41598-019-49737-2

**Published:** 2019-09-11

**Authors:** Qingsong Bai, Yanling Cai, Boxiang He, Wanchuan Liu, Qingyou Pan, Qian Zhang

**Affiliations:** 1 Guangdong Provincial Key Laboratory of Silviculture, Protection and Utilization, Guangdong Academy of Forestry, Guangzhou, 510520 China; 2 Guangdong Academy of Forestry, Guangzhou, 510520 China; 3 Xinyi Forestry Research Institute, Maoming, 525300 China

**Keywords:** Plant breeding, Plant molecular biology

## Abstract

Germplasm resource collection and utilization are important in forestry species breeding. High-through sequencing technologies have been playing increasing roles in forestry breeding. In this study, specific-locus amplified fragment sequencing (SLAF-seq) was employed to analyze 149 masson pine (*Pinus massoniana*) accessions collected from Guangdong in China. A large number of 471,660 SNPs in the total collection were identified from 599,164 polymorphic SLAF tags. Population structure analysis showed that 149 masson pines could not be obviously divided into subpopulations. Two core sets, containing 29 masson pine accessions for increasing resin and wood yield respectively, were obtained from the total collection. Phenotypic analyses of five traits showed abundant variations, 25 suggestive and 9 significant SNPs were associated with the resin-yielding capacity (RYC’) and volume of wood (VW) using EMMAX and FaST-LMM; 22 suggestive and 11 significant SNPs were associated with RYC’ and VW using mrMLM and FASTmrMLM. Moreover, a large number of associated SNPs were detected in trait HT, DBH, RW and RYC using mrMLM, FASTmrMLM, FASTmrEMMA and ISIS EM-BLASSO. The core germplasm sets would be a valuable resource for masson pine improvement and breeding. In addition, the associated SNP markers would be meaningful for masson pine resource selection.

## Introduction

Masson pine (*Pinus massoniana*) is a native species that grows throughout central and southern China. Besides its wide uses in the wood, pulp and paper industries, this species has long been employed as the main source of resin, a hydrocarbon secretion of many plants that is widely used to produce resin and turpentine for the chemical industry^1^. Masson pine is the most important resin tapping tree species in China and should thus be preserved^2^. However, due to its high commercial value, this species has been subjected to over-exploitation during past decades, leading to a gradual decrease in genetic resources^3^. Protection and sustainable use of the preserved masson pine resource are urgent problems for researchers.

Genetic structure and diversity analyses could help to scientifically simplify the resources. Various types of molecular markers, including RAPD, SRAP, SSR, and ISSR, have been used to estimate genetic relationships and genetic distances in masson pine^4–9^. Single nucleotide polymorphisms (SNPs) have been widely reported in recent years because they are the most abundant and stable type of genetic marker in most genomes^10^. Deep sequencing technology has been rapidly developed to exploit these advantages and has enabled the high-throughput identification of SNPs^11–13^, albeit with the disadvantage of becoming cost-prohibitive when the population is large. The genomes of conifer trees such as *Pinus taeda* are complex and fairly long^14^. To reduce time and labor costs, reduced-representation genome sequencing has been widely used in plant genome sequencing^14^. Considering that whole-genome deep sequencing is still expensive and usually unnecessary^11^, several simplified and cost-effective methods for SNP discovery and high-throughput genotyping have been developed, such as reduced representation library (RRL) sequencing^15^, restriction-site associated DNA sequencing (RAD)^16,17^, and two-enzyme genotyping by sequencing (GBS)^18^. In recent years, a new strategy for de novo SNP discovery and genotyping of large populations, referred to as specific-locus amplified fragment sequencing (SLAF-seq), has been employed^19^. SLAF-seq is a high throughput, highly fast, highly efficient and cost-effective method for developing large-scale SNP and InDel markers^19^. By using enzyme digestion techniques, an SLAF-seq library containing specific size fragments of DNA can be obtained. Then, we could identify a polymorphic specific SNP locus from all of the accessions through software alignment. This high-resolution method has been tested on many organisms, including crape myrtle^20^, cucumber^21^, rapeseed^22^, sesame^23^ and soybean^24^. Moreover, this method has been widely used in GWAS for important traits^20,25,26^, as well as in the development of core germplasm^27^.

To better understand the genetic relationship and the genetic architecture of wood and resin yield traits of the *P. massoniana* accessions in Guangdong province, we conduct a genome-wide SNP discovery based on the SLAF-seq method. The identified SNPs were used to examine the masson pine population structure. Then, we selected a core set of masson pine germplasm resources for improving resin-yielding capacity (RYC) and volume of wood (VW). Finally, a genome-wide association study (GWAS) strategy was used to identify the SNP locus associated with growth, wood and resin yield traits. The results would be of great value for masson pine selection and breeding.

## Results

### Sequencing quality statistics

By SLAF-seq, 1759.00 M reads were obtained from this experiment. The average Q3 value was 92.78%, and the average GC content was 37.95% (see Supplementary Table S1). A large number of 3,232,864 SLAF tags were identified throughout the masson pine genome. The average sequencing depth of the tags was 16.98× (see Supplementary Table S2). Subsequently, a total of 599,164 polymorphic SLAF tags containing 2,774,976 SNPs were developed for the 149 samples that were used for further analysis. After filtering out the invalid SNPs, 471,660 SNPs were remained among the 149 masson pine accessions.

### Population structure and linkage disequilibrium analysis

**Figure 1:**
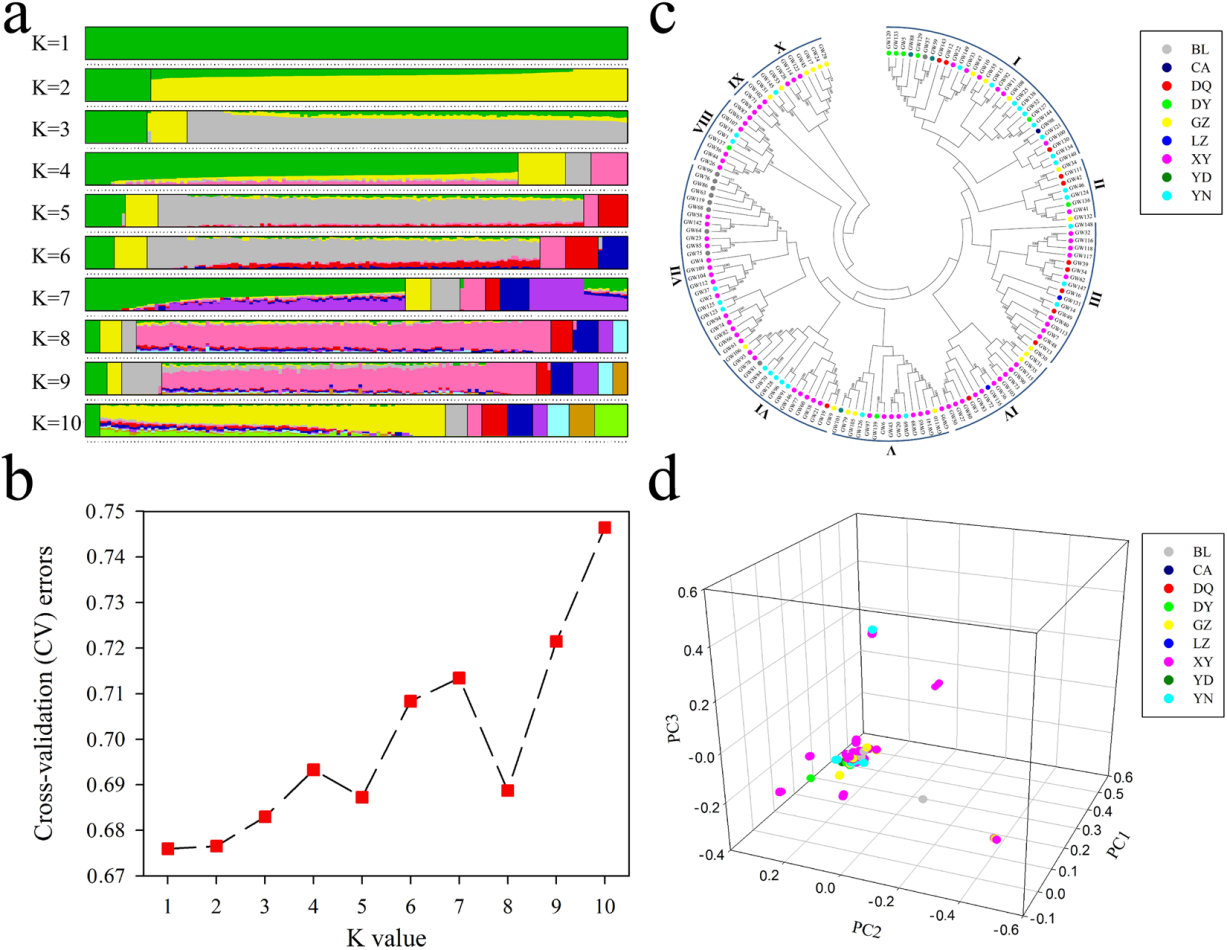
Population structure, validation, phylogenetic and PCA of 149 masson pine accessions. (**a**) The population structure. The x-axis indicates different accessions. The y-axis quantifies the membership probability of accessions belonging to different groups. Colors in each row represent structural components. (**b**) The ADMIXTURE estimation of the number of groups for K values ranging from 1 to 10. The K value with the lowest CV error represents the suggested cluster number. (**c**) The phylogenetic tree of 149 masson pines was built by the neighbor-joining method with 1000 bootstrap replications in MEGA 6.0 software. Roman numbers indicate the subgroups. (**d**) The principal component analysis (PCA) of 149 masson pine accessions.

We applied clustering analysis to the samples using ADMIXTURE software (Fig. 1a). This method has been used with large sample sizes, exhibiting a strong capability to assign individuals into populations. The estimated membership fractions of the 149 accessions for different values of K ranged from 1 to 10, and the maximum likelihood revealed by the population structure showed an optimum value of 1 (K = 1; Fig. 1b), indicating that the masson pines in Guangdong could not be categorized into different subpopulations. It is important to use population-based methods to separate accessions from mixed populations into unstructured subpopulations, as this allows for association analyses between phenotypes and molecular bands to be conducted in homogeneous subpopulations^28^. Population analysis indicated that these masson pines were not excessively separated and could be used for association analysis. We also used the structure and fastStructure to calculate appropriate K value (see Supplementary Fig. S1). The results showed that the highest delta K value was obtained when K of the masson pine population was 2; the highest marginal likelihood was obtained when the K value was 6. The geographical distributions of the masson pines were also not consistent with the population structure in the two methods. Some more discussions should be added in the population structure analysis of masson pines in Guangdong.

Linkage disequilibrium (LD) is the non-random association of alleles at different loci and may indicate the genetic forces that structure the genome^29^. Investigations of genetic diversity and LD are prerequisites for association; both aid in the interpretation of results. LD estimates in this study based on the specific length sequences indicated a very fast decay. A collection of 515,555,111 pairwise comparisons with relatively high LD (r^2^ ≥ 0.10) was found among the above mentioned SNPs (see Supplementary Fig. S2). The majority of the LD estimates (97.0%) presented an r^2^ value lower than 0.50 (0.10–0.50); only 3.0% displayed very high LD (0.51–1.00). Mean r^2^ values of conifers differ among different species^30^. The generality of LD distribution across the entire masson pine genome remains to be further analyzed, as only a relatively small part of the entire genome was studied here.

### Genetic relationship analysis

Based on the analysis of 471,660 SNPs, a neighbor-joining tree was constructed using MEGA software (Fig. 1c). The 149 masson pine accessions were divided into ten subgroups by the neighbor-joining analysis. In general, the genetic relationships among these masson pines were not consistent with their geographical distributions. Most subgroups had masson pines from more than three districts. In subgroup VII, masson pines in the west (XY) clustered with the masson pines in the east (BL). However, their geographic locations showed a relatively far distance. Similar phenomena also happened in subgroup I and VI. This indicated that the masson pines in Guangdong may be closely related. Relationship coefficients between the 149 samples were calculated (see Supplementary Fig. S3). Of the 22,052 pairwise combinations, 21,895 (99.29%) had genetic relationship coefficients <0.05. Only a very small fraction of pairwise combinations had genetic relationship coefficients >0.05. PCA was performed using the same SNPs to estimate the clusters within the population (Fig. 1d). The PCA result was consistent with the assignments made using ADMIXTURE, i.e., there was one main group and several smaller groups with a small quantity of members. Some masson pines appeared to be separated from the main group. This may have been due to the uneven variation in the population. Masson pines distributed in nine regions in Guangdong province were intermixed, indicating that the masson pines in Guangdong may derive from the same provenance. However, from the phylogenetic tree made by MEGA, all of the masson pine accessions could be categorized into 10 subgroups, which meant that there were also major distinctions among masson pines in Guangdong.

### Development of core germplasm sets

**Figure 2:**
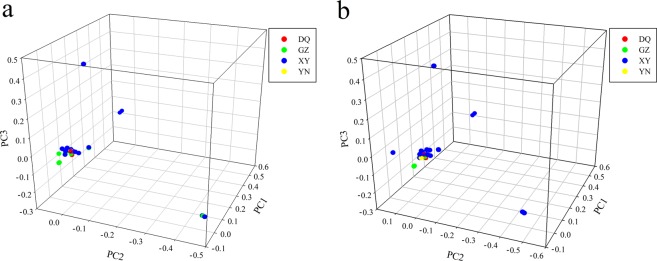
The PCA plots of core germplasm sets for resin and wood. (**a**) The PCA plot of the core set for resin. (**b**) The PCA plot of the core set for wood.

Genetic distance was estimated to evaluate the genetic diversity in all the accessions. Masson pine accessions GW29 and GW28 had the highest genetic distance (0.292). GW37 and GW112 had the lowest genetic distance (0.008). Genetic distance and population structure were used to select core germplasms. In this study, core sets containing 29 accessions were screened out and combined with traits VW and RYC, respectively (Table 1), including 24 common accessions for both wood and resin. The 29 accessions were derived from four regions (DQ, GZ, XY, and YN). Genetic distance and population structure were analyzed for the core set. The mean genetic distance in the core set of resin was 0.237 and ranged from 0.015 to 0.277; the mean genetic distance in the core set of wood was 0.236 and ranged from 0.0165 to 0.273. The mean genetic distances of both core germplasm sets were higher than that of the total collection (0.232). The core set PCA plots of resin and wood also showed a similar structure with the total collection (Fig. 2). These masson pine lines were genetically and geographically distantly distributed. Hence, the core germplasm population is an upgraded collection for breeding and could be available for distant hybridization in the future.

**Table 1:** Phenotypes and categories of core sets for wood and resin.

Accession	Location	VW	RYC	Core set for wood	Core set for resin
GW54	DQ	0.42	342.56	◯	◯
GW111	DQ	0.76	302.02	◯	◯
GW9	GZ	0.28	177.93	◯	◯
GW24	GZ	0.45	235.84	◯	◯
GW29	GZ	0.31	193.82	◯	◯
GW31	GZ	0.3	206.39	◯	◯
GW45	GZ	0.3	220.82	◯	◯
GW30	GZ	0.26	213.42	◯	
GW51	GZ	0.26	184.64	◯	
GW106	GZ	0.22	192.44	◯	
GW2	XY	0.3	252.16	◯	◯
GW4	XY	0.32	202.59	◯	◯
GW7	XY	0.34	225.14	◯	◯
GW8	XY	0.42	248.18	◯	◯
GW20	XY	0.29	237.42	◯	◯
GW23	XY	0.35	260.06	◯	◯
GW32	XY	0.31	186.59	◯	◯
GW36	XY	0.32	204.42	◯	◯
GW48	XY	0.29	201.89	◯	◯
GW71	XY	0.37	237.71	◯	◯
GW72	XY	0.44	238.97	◯	◯
GW78	XY	0.44	301.81	◯	◯
GW85	XY	0.32	180.46	◯	◯
GW93	XY	0.33	254.26	◯	◯
GW116	XY	0.38	191.65	◯	◯
GW118	XY	0.39	255.66	◯	◯
GW27	XY	0.26	184.92	◯	
GW50	XY	0.28	218.07	◯	
GW87	XY	0.42	186.79		◯
GW103	XY	0.39	168.39		◯
GW109	XY	0.3	164.84		◯
GW142	XY	0.33	176.77		◯
GW91	YN	0.5	192.2	◯	◯
GW52	YN	0.32	170.98		◯

### Association analysis of growth and economic traits

In total, a set of 122 masson pine accessions, including various levels of height (HT), diameter at breast height (DBH), resin weight (RW), volume of wood (VW), and resin-yielding capacity (RYC) were used for association analysis. In addition, 69 clonal lines were employed for RYC’ association analysis. The average value and standard deviation of six traits are listed in Supplementary Fig. S4. We also calculated the frequency distributions of the phenotypic data. The results showed that HT, DBH, VW, RW, and RYC were normally distributed. In addition, these data indicated a high degree of diversity in phenotypic traits in the population. The frequency of trait RYC’ was not a complete normal distribution, but it also demonstrated a high degree of diversity. Hence, these phenotype data would be used for genome-wide association analysis.

**Figure 3:**
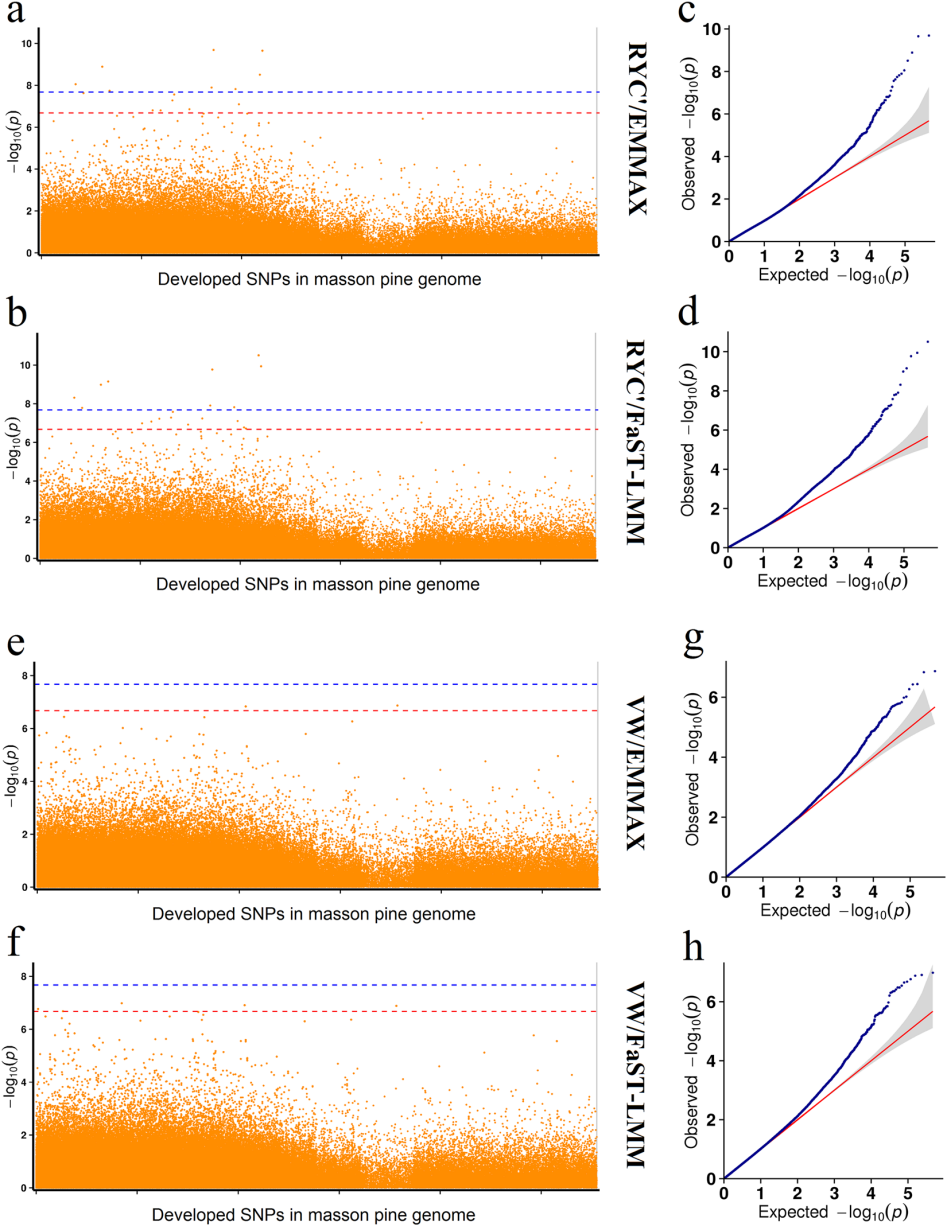
The manhattan plots and Q–Q plots of traits RYC’ and VW using EMMAX and FaST-LMM. Each dot in the Manhattan plot represents one SNP. The horizontal dotted red and blue lines indicate the suggestive and significant thresholds.

The GWAS was performed using SNPs and phenotypic data. In our study, a total of 472,348 SNPs remained in the 122 accessions, and 476,264 SNPs remained in 69 accessions after filtering out of the invalid SNPs. Thus, the genome wide significant and suggestive P-values in 122 accessions were 2.12 × 10^−8^ (0.01/472,348) and 2.12 × 10^−7^ (0.1/472,348), respectively. Among the 69 accessions, the P-values were 2.10 × 10^−8^ (0.01/476,264) and 2.10 × 10^−7^ (0.1/476,264). The GWAS analysis was carried out by the methods of MLM, FaST-LMM, EMMAX, mrMLM, FASTmrMLM, FASTmrEMMA, ISIS EM-BLASSO, pKWmEB and pLARmEB. The results showed that some SNPs were detected and associated with VW and RYC’ by multiple methods. A total of 15 RYC’ associated SNPs and 2 VW associated SNPs were developed using EMMAX method (Fig. 3a,e), 20 RYC’ associated SNPs and 5 VW associated SNPs were developed using FaST-LMM method (Fig. 3b,f). In addition, a total of 9 and 8 significant SNPs were developed by method FaST-LMM and EMMAX in trait RYC’. It is interesting that the SNPs developed by the EMMAX method completely overlapped with the SNPs developed by the FaST-LMM method irrespective of trait RYC’ or VW (Table 2). Eight SNPs (Marker643442, Marker650102, Marker530780, Marker297054, Marker279561, Marker210060, Marker526082, Marker582947) that significantly associated with RYC’ were simultaneously developed by EMMAX and FaST-LMM methods, which indicated that those SNPs were very valuable and significant in breeding. However, no SNPs were developed by MLM methods in all the traits.

**Table 2:** RYC’ and VW associated SNPs using EMMAX and FAST-LMM.

Trait	Marker	Position	Alleles	EMMAX	P-value	FaST-LMM	P-value
RYC’	Marker643442	96	T/C	◯	3.12E-09	◯	3.12E-11
	Marker650102	85	A/G	◯	2.22E-10	◯	1.15E-10
	Marker530780	30	G/T	◯	2.06E-10	◯	1.70E-10
	Marker297054	111	T/G	◯	1.82E-08	◯	7.07E-10
	Marker279561	86	G/A	◯	1.30E-09	◯	1.04E-09
	Marker210060	123	C/T	◯	8.90E-09	◯	4.87E-09
	Marker526082	61	C/T	◯	1.28E-08	◯	1.24E-08
	Marker582947	199	G/A	◯	1.52E-08	◯	1.51E-08
	Marker231539	255	T/G	◯	2.37E-08	◯	1.63E-08
	Marker441368	48	C/T	◯	2.76E-08	◯	2.63E-08
	Marker437366	105	C/T	◯	5.29E-08	◯	5.23E-08
	Marker507605	70	C/A			◯	5.81E-08
	Marker411469	183	T/C	◯	1.58E-07	◯	5.96E-08
	Marker591100	241	G/C	◯	8.08E-08	◯	7.84E-08
	Marker394053	201	G/A	◯	1.57E-07	◯	8.36E-08
	Marker1560613	202	T/C			◯	9.35E-08
	Marker373316	73	C/T			◯	1.03E-07
	Marker475399	42	C/T	◯	1.38E-07	◯	1.20E-07
	Marker607165	200	T/C			◯	1.68E-07
	Marker611740	8	G/T			◯	1.91E-07
VW	Marker335582	178	T/C			◯	1.04E-07
	Marker613704	194	C/T	◯	1.47E-07	◯	1.23E-07
	Marker1179037	157	G/C	◯	1.35E-07	◯	1.31E-07
	Marker103424	115	C/T			◯	1.71E-07
	Marker188304	115	G/A			◯	2.09E-07

Note:◯means that the SNP could be developed by that model.

**Figure 4:**
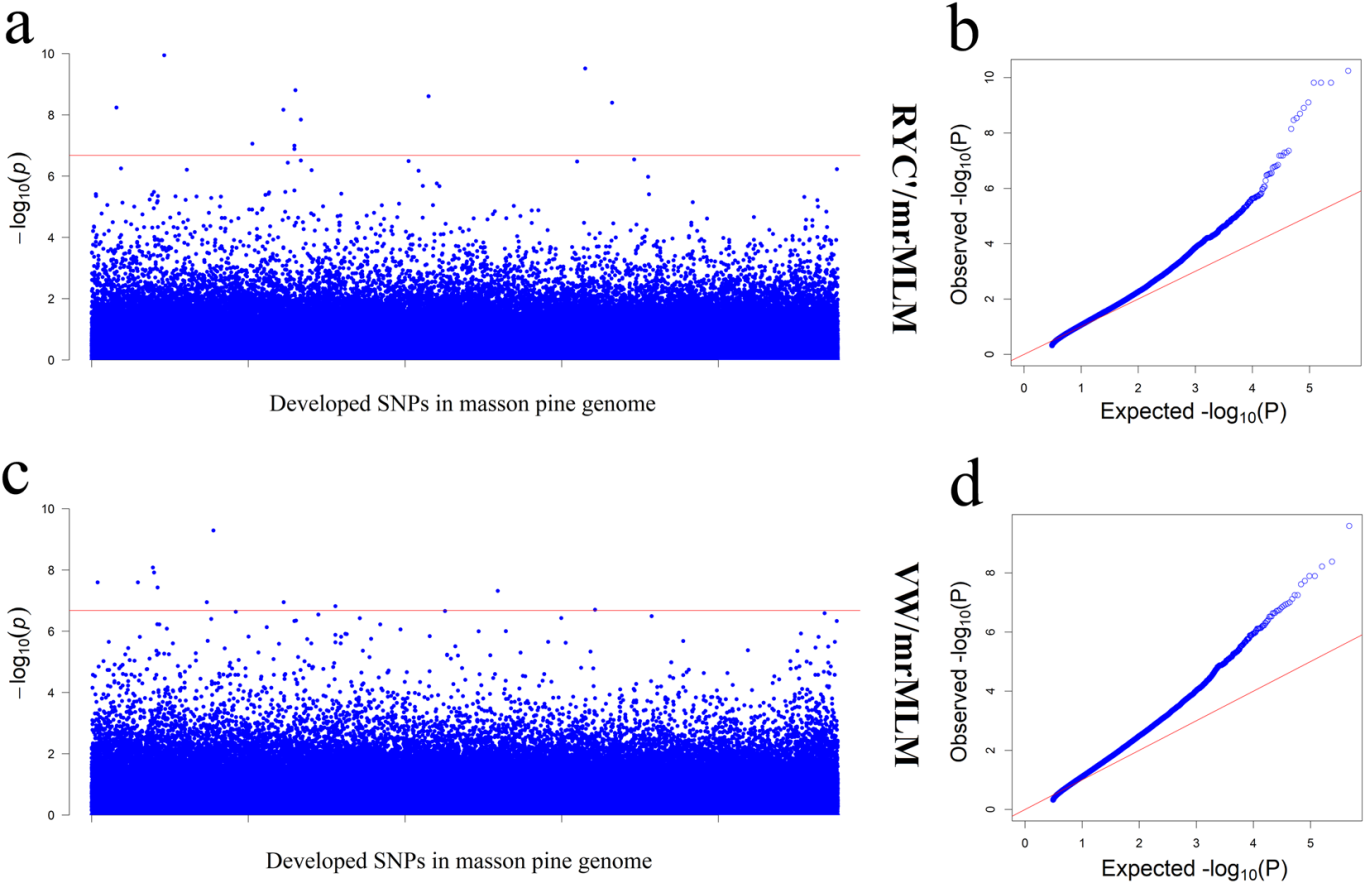
The manhattan plots and Q–Q plots of traits RYC’ and VW using mrMLM. The horizontal dotted red lines indicate the suggestive thresholds.

In this study, we also used the multi-locus methods mrMLM, FASTmrMLM, FASTmrEMMA, ISIS EM-BLASSO, pKWmEB and pLARmEB in mrMLM.GUI version 3.2 to identify associated SNPs. The result showed that 11 SNPs and 11 SNPs were associated with trait RYC’ and VW using mrMLM method, including 8 significant SNPs (Marker124737, Marker174624, Marker482425, Marker279561, Marker370341, Marker504406, Marker271387 and Marker283415) associated with trait RYC’ and 3 significant SNPs (Marker217315, Marker163256 and Marker164392) associated with trait VW (Fig. 4 and Table 3). The associated SNPs developed by FASTmrMLM were totally identical to the SNPs developed by mrMLM according to the P-value. We did not obtain associated SNPs from FASTmrEMMA, ISIS EM-BLASSO, pKWmEB and pLARmEB. After comparing the differences among the SNPs developed by different methods, we found that Marker279561 was simultaneously developed by methods EMMAX, FaST-LMM, mrMLM, FASTmrMLM and ISIS EM-BLASSO. The remaining SNPs developed by methods mrMLM and FASTmrMLM were different from methods EMMAX and FaST-LMM.

**Table 3:** RYC’ and VW associated SNPs using mrMLM.

Trait	Marker	Position	Alleles	mrMLM	P-value	FASTmrMLM	P-value
RYC’	Marker174624	234	C/T	◯	1.14E-10	◯	1.14E-10
	Marker482425	206	C/T	◯	3.06E-10	◯	3.06E-10
	Marker279561	86	G/A	◯	1.57E-09	◯	1.57E-09
	Marker370341	258	C/T	◯	2.46E-09	◯	2.46E-09
	Marker504406	117	C/G	◯	4.04E-09	◯	4.04E-09
	Marker124737	43	G/T	◯	5.79E-09	◯	5.79E-09
	Marker271387	80	A/G	◯	6.79E-09	◯	6.79E-09
	Marker283415	234	C/T	◯	1.42E-08	◯	1.42E-08
	Marker248105	192	G/T	◯	8.77E-08	◯	8.77E-08
	Marker278935	7	C/T	◯	1.01E-07	◯	1.01E-07
	Marker278935	256	A/C	◯	1.31E-07	◯	1.31E-07
VW	Marker217315	18	G/A	◯	5.16E-10	◯	5.16E-10
	Marker163256	68	C/A	◯	8.3E-09	◯	8.3E-09
	Marker164392	258	T/C	◯	1.21E-08	◯	1.21E-08
	Marker103247	160	T/C	◯	2.55E-08	◯	2.55E-08
	Marker147979	68	T/G	◯	2.55E-08	◯	2.55E-08
	Marker167996	196	A/G	◯	3.74E-08	◯	3.74E-08
	Marker417759	73	G/A	◯	4.83E-08	◯	4.83E-08
	Marker211769	144	G/A	◯	1.13E-07	◯	1.13E-07
	Marker271496	74	C/G	◯	1.13E-07	◯	1.13E-07
	Marker308123	258	T/G	◯	1.52E-07	◯	1.52E-07
	Marker490183	52	C/T	◯	1.99E-07	◯	1.99E-07

**Figure 5:**
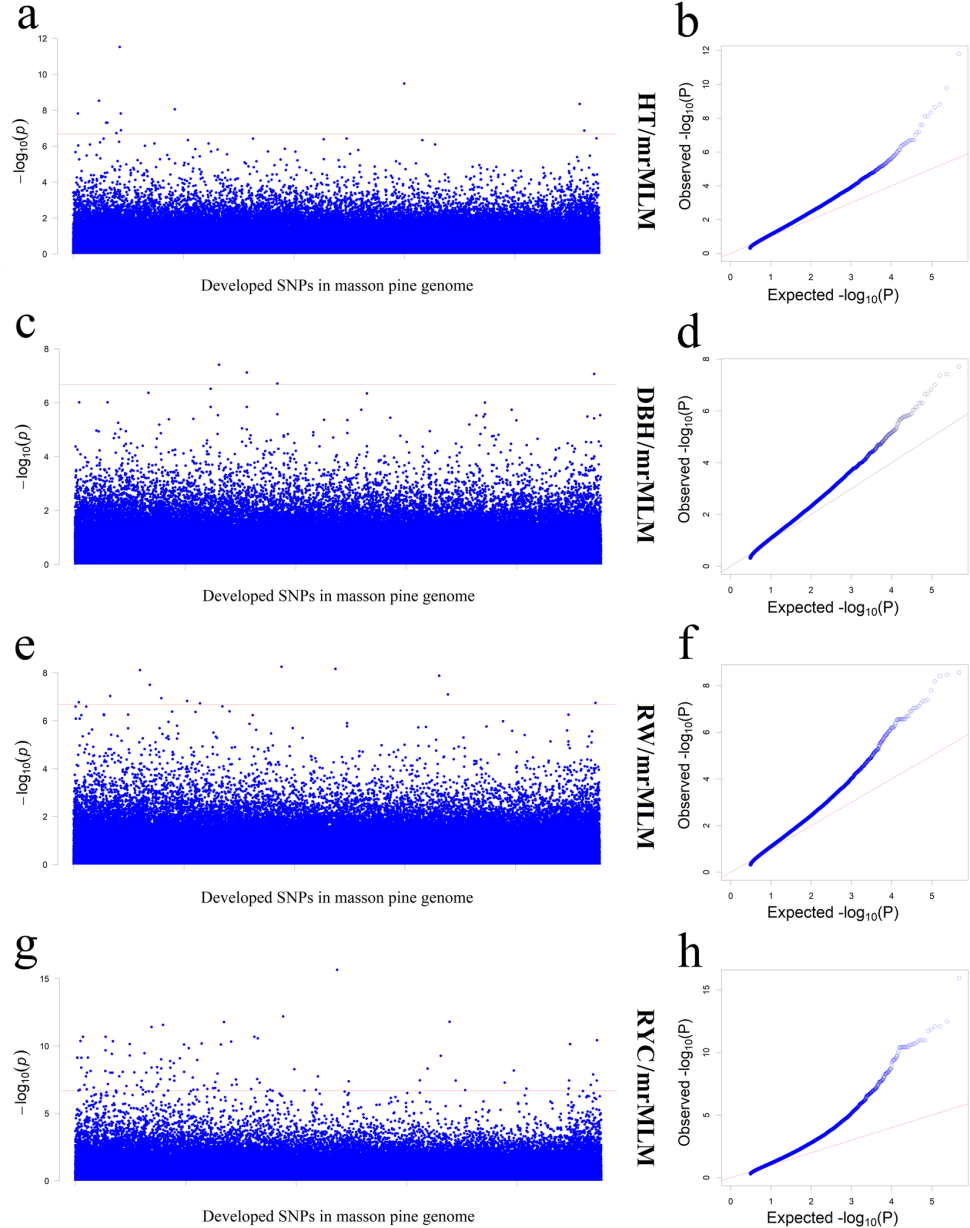
The manhattan plots and Q–Q plots of traits HT, DBH, RW and VW using mrMLM. The horizontal dotted red lines indicate the suggestive thresholds.

According to the association results of trait HT, DBH, RW and RYC, 12, 4, 14 and 136 suggestive SNPs were developed using the methods in procedure mrMLM (see Fig. 5 and Supplementary Table S3). A total of 6, 4 and 76 significant SNPs were developed in trait HT, RW and RYC. It is interesting that all the developed SNPs in trait RW were simultaneously developed in trait RYC. According to the LOD value, 5, 4, 2, 2 and 1 SNPs were significantly associated (LOD ≥ 3) with trait RYC’, VW, HT, DBH and RW by at least two methods (see Supplementary Table S4).

### Gene identification of associated SNPs

Until now, there is no available public *P. massoniana* genome database on the website. We screened out the SNPs located SLAF sequences. After BLASTN analysis with public database, most results were located on the non-coding regions in genomic DNA. Among all the associated SNPs, 26 SLAF sequences were directly located on the conserved domain of functional genes (see Supplementary Table S5). The genes were involved in RNase_H_like super family, RT_like super family, RVT_2 super family, FusA super family, pepsin_retropepsin_like super family, ribokinase_pfkB_like super family and rve super family.

## Discussion

It takes a long time to evaluate the growth and economic traits in a conventional breeding program, and markers that facilitate selection of trees with high yields of resin and wood will have a major impact on masson pine breeding^13^. Traditional breeding methods are usually inefficient for forestry species. In recent years, genomic data have provided research workers novel insight into *P. massoniana* genetic diversity and evolution^5,7,9,31,32^. With the development of next-generation sequencing, sequencing technologies, such as GBS, RAD-seq, and SLAF-seq, are now available for the identification of abundant SNPs in a wide range of plant species^17,22^. Therefore, development of SNP markers using SLAF-seq can currently meet the needs of GWAS in masson pine^19^. In this study, 149 masson pine accessions collected from different regions of Guangdong in China were employed to develop SLAFs for SNP detection. The genetic structure of diverse masson pine accessions was estimated using 471,660 SNPs. Long-term selection gain of forestry trees requires large numbers of resources with genetic variability. Therefore, the examination of the population structure and genetic diversity are both important for the a breeding program^33^. In this study, we received different population numbers using cross-validation, delta K analysis and fastStructure analysis. The cross-validation support the result of K = 1. Moreover, the result of PCA also indicated one principal component with several separated individuals. The clustering analysis also did not show obvious separations. Through the clustering analysis, some masson pine accessions from far geographical distances clustered in the same subgroup, which indicated that the masson pines in Guangdong are not genetically distantly related. Guangdong is surrounded by numerous mountains and has an independent geographical environment. Thus, the gene exchanges of masson pines may be limited in the whole province. Furthermore, masson pine has a long period of cultivation history; the breeding work and provenance tests started in Guangdong province in the last century. The plus families of masson pine were planted across Guangdong province. The intermixed relationships among some masson pines collected from different regions may be induced by the cultivation history. By using clustering analysis, the masson pines were divided into different subgroups by the genetic distance, which meant that there are great differences among these germplasm resources. In future study, masson pines derived from other regions should be collected and compared to the current resources.

Molecular markers have been employed to develop core germplasm sets in multiple tree species, e.g. western white pine^34^, olive^28,35^, litchi^36^, pear^37^, and Chinese fir^38^ have been examined using SNPs developed by reduced-representation genome sequencing. A core set percentage of 20~30% of the total collection was once suggested at a general scale of the population^27^. The fixed size of the core set depends on the purpose of the study, and different kinds of plants require different sampling percentages^39^. Long-term selection gain requires genetic variability; thus, it is important to examine not only population structure but also genetic diversity^33^. Across the 149 masson pine accessions examined in this study, we observed a mean genetic distance of 0.232, with a range from 0.008 to 0.292. Furthermore, genomic characterization revealed high genetic diversity within the 149 masson pine accessions; therefore, we decided to identify a core germplasm set to improve masson pine breeding efficiency. It is important and meaningful to select a fully representative germplasm set from a large masson pine collection. In this study, the core sets of wood and resin showed higher genetic distances than the total collection. In addition, the core set of wood showed a high level of genetic gain expectation (41.78%) for trait VW; the core set of resin showed a high level of genetic gain expectation (40.75%) for trait RYC. The core germplasm sets, for the purpose of improving resin and wood yield, were scientifically simplified resources that would be useful for masson pine breeding.

The GWAS analyses of complex traits in forestry conifer trees, especially conifer trees with large genomes, require an enormous density of SNP markers^40^. The decay of LD over physical distances in a population determines the density of the marker coverage needed to perform a GWAS^41^. The faster LD decays, the more markers are likely needed in GWAS analysis for complex traits. LD estimates in this study based on the specific length sequences indicated a very fast decay. Excavation of favorable markers is necessary for improving masson pine breeding efficiency using molecular assisted selection (MAS). GWAS offers increased opportunities for detecting susceptible loci for complex traits. Masson pine is an economic tree species for resin and wood. In the breeding project of masson pine, both resin and wood yields are important breeding targets. Therefore, discovering SNPs related to resin and wood producing capacity is important for improving masson pine breeding efficiency.

In the present study, we focused on the GWAS of quantitative traits, including growth traits and the resin and wood yield in masson pine. The phenotypes of complex traits often result from the combined actions of multiple genes and environmental factors, all of which can easily lead to lost heritability^42^. Therefore, only those traits with high heritability can be stably detected. The traits in masson pines, especially RYC and VW, have been demonstrated to have high heritability^43^. Furthermore, more extensive linkage disequilibrium has been found in conifer trees^44,45^. In our study, the number of SNPs identified from 149 masson pine germplasm resources is large enough, and GWAS can be feasible in masson pine even though the genome may be generally large^13^. RYC and VW are important traits for representing masson pine producing capacity and economic value and have an important value in breeding^43^. In this study, five traits (HT, DBH, RW, VW, and RYC) were selected for GWAS analyses. All of the traits showed large phenotypic variation, supporting the suitability of GWAS for these traits. Thus, we presented GWAS analyses of these important traits in masson pine. In our study, the suggestive SNPs associated with traits RYC’ and VW were different from the SNPs identified using EMMAX and FaST-LMM. Only one common SNP (Marker279561) was developed in trait RYC’ and VW using these methods, which meant that different types of GWAS methods can provide complementary results with each other and provide us with more sufficient results. Moreover, no SNPs were developed in trait HT, DBH, RW and RYC using EMMAX and FaST-LMM, while a large number of SNPs were detected using multi-locus methods in mrMLM. The multi-locus GWAS methods in mrMLM.GUI provide more possibilities in detecting associated SNPs. Hence, a group of various types of GWAS methods should be applied in future studies.

High correlations between these traits were identified, and strong positive correlations existed among the traits DBH, RW, VW, and RYC (see Supplementary Table S6). The SNPs developed in trait RW were totally detected in trait RYC which meant that these SNPs have significance in the selection of high resin yield masson pines. However, the other trait did not show correlation ships, indicating that it is also necessary to develop additional SNPs at higher levels in the future. In recent years, MAS and genome selection (GS) have been the most popular methods in plant breeding^46,47^. GWAS and GS can each compensate for the other’s deficiencies, and both approaches are likely to be useful in conifer breeding. The developed SNP markers in GWAS can be directly used for both MAS and GS, and both approaches are likely to be useful in conifer breeding. Genotyping based on reduced-representation genome sequencing (RRGS) has become popular in a wide range of plant species^17,48^. The various types of RRGS methods, among which SLAF-seq is also widely used, have overcome the cost problem and have simplified the problem of identifying a large number of DNA markers in conifer species with large genomes as well as the large number of samples in the scientific research of forestry breeding.

*P. massoniana* has not been completely genome sequenced. By using BLASTN with the public database and conserved domain search, several SNPs were located on the conserved domain in some unusual genes. The other SNPs were mainly distributed on the noncoding region of genome DNA. Further annotations and functional analysis of those SNPs are necessary. Future studies of masson pine should not merely focus on RRGS methods, a various types of methods such as exon capturing and comparative transcriptome sequencing should be also considered for detecting SNPs and functional genes. The SNPs developed from exon-seq and RNA-seq are usually distributed on the transcript sequences and has been successfully used in conifer species^49^.

## Conclusion

In this study, SLAF-seq technology was used to develop 471,660 filtered SNPs from 149 *P. massoniana* accessions in Guangdong. The population structure and genetic relationship analyses of these masson pines showed a chaotic genetic relationship but various genetic distances. We obtained core germplasm sets including 29 masson pine accessions for increasing wood and resin production, respectively. Multiple methods were used in GWAS of five traits and the results provided us different associated SNPs. The application of various GWAS methods can enrich the number of associated SNPs. The core germplasm resources and identified SNPs have meaningful application values in *P. massoniana* selection and breeding.

## Materials and Methods

### Experimental materials

A total of 149 masson pine accessions were selected for obtaining SNP markers (see Supplementary Table S1). The masson pines were collected from Boluo (BL), Chaoan (CA), Deqing (DQ), Dongyuan (DY), Gaozhou (GZ), Lianzhou (LZ), Xinyi (XY), Yingde (YD), and Yunan (YN) in Guangdong province in southern China; in the latitude 21°55′N– 23°87′N, longitude 110°47′E– 114°41′E, and at elevations from 35 m to 458 m. Those lines were planted in a masson pine seed orchard in 1989 by the grafting method. For each accession, 0.5 g of clean conifer needles was selected from each accession for further DNA extraction.

### DNA extraction and SLAF-seq

Total masson pine genomic DNA was extracted using the DP320 DNA secure Plant Kit (TIANGEN China); the quality and quantity of DNA were then inspected using 0.8% gel electrophoresis. The quantified DNA was diluted to 20 μg·μL^−1^ and was stored at −20 °C before use. The masson pine genomic DNA was analyzed according to the SLAF-seq method^19^. To obtain evenly distributed SLAF tags and to avoid repetitive SLAF tags for maximum SLAF-seq efficiency, simulated restriction enzyme digestion was carried out in silico. Sequencing libraries of each accession were constructed through digestion with the restriction enzymes EcoRV and ScaI to obtain the SLAF tags, and *Oryza sativa* genome DNA was used as a control to assess the normal rate of enzyme digestion. A single nucleotide (A) overhang was added to the digested fragments using dATP at 37 °C, and then duplex tag-labeled sequencing adapters were ligated to the A-tailed DNA with T4 DNA ligase. The PCR products were purified and pooled. The pooled samples were separated via electrophoresis on a 2% agarose gel. Fragments with indices and adaptors from 264 to 414 bp were excised and purified. Finally, the purified gel product was sequenced using the Illumina HiSeq2500 system (Illumina, Inc., San Diego, CA, USA) at the Biomarker Technologies Corporation in Beijing.

### Genotyping and quality control

After sequencing, reads with double ends were compared with similar sequences that could be labeled as candidate SLAFs to proceed with the next step. The SLAF tags were defined as the group with the most samples. The samples with the most tags were used as references, and GATK and SAMTOOLS were employed for SNP calling^50,51^. SNPs were removed if the integrity <0.8 and minor allele frequency (MAF) ≤0.05. After these steps, the remaining SNPs were developed to calculate genetic structure, and the relationships were retained for genome-wide association study (GWAS).

### Structure, phylogenetic and genetic kinship among accessions

SNPs were used to calculate pairwise kinship relationships among the 149 accessions by using SPAGeDi software^52^. Negative kinship values between two accessions indicate a poorer relationship than expected, and this was corrected to 0^53^. ADMIXTURE was employed to investigate population structure based on the maximum-likelihood method^54^. The predefined K, which indicates the number of groups in a population, varied from 1 to 10 in ADMIXTURE models. Cross-validation, delta K and marginal likelihood against K were used to select the most probable value of K^55^. A phylogenetic tree based on the neighbor-joining method was constructed in MEGA 6.0 using the developed SNPs^56^. A PCA with Cluster software was used to cluster the masson pine population^57^. Genetic distance and population structure were used to develop an initial core germplasm set by CoreHunter software^58^. The results combined with phenotypic data VW and RYC were used to confirm the final core set.

### Phenotypic data collection and analysis

Phenotype data, including height (HT), diameter at breast height (DBH), resin weight (RW), volume of wood (VW) and resin-yielding capacity (RYC), of 122 lines from 605 clone individuals were measured in 2010. RYC’ data were collected and calculated from 69 plus trees. Firstly, we collected the phenotype data of individuals. Then, the average value of individuals from the same accession was used as the final phenotype data. Resins were collected on sunny days from July to October using the narrow face system as described by COPPEN and HONE^1^. Trees were sampled once per day by removal of a sliver of wood from the stem without the application of a stimulant. VW and RYC were calculated by the formulas given below.

The VW of an individual was calculated as follows^43^:

1 $${\rm{VW}}=6.2341803\times {10}^{-5}\times {{\rm{DBH}}}^{1.8551497}\times {{\rm{HT}}}^{0.95682492},$$

where: VW is the volume of wood from an individual tree; DBH is the diameter at breast height in meters, and HT is the height of the tree in meters.

The RYC of an individual was calculated as follows^43^:

2 $${\rm{RYC}}=\frac{{\rm{Wt}}}{({\rm{D}}\times {\rm{Wd}}/{\rm{C}})},$$

where: RYC is the resin-yielding capacity of an individual tree; Wt is the total weight of collected resin of a tree; D is the cutting time for resin tapping per tree; Wd is the total width of the narrow tapping face; and C is the circumference of the trunk where the bark was cut.

### Association analysis

The GWAS analysis was performed by multiple methods, namely, the Mixed Linear Model (MLM) in TASSLE software^59^, Factored Spectrally Transformed Linear Mixed Models (FaST-LMM) in FaST-LMM software^60^, Efficient Mixed-Model Association eXpedited (EMMAX) in EMMAX software^61^ and six methods, including multi-locus random effect mixed linear model (mrMLM)^62^, fast Multi-locus random effect mixed linear model (FASTmrMLM)^63^, fast multi-locus random-SNP-effect EMMA (FASTmrEMMA)^64^, iterative modified-sure independence screening Expectation-Maximization-Bayesian least absolute shrinkage and selection operator (ISIS EM-BLASSO)^65^, polygenic-background-control-based Kruskal-Wallis test with empirical Bayes (pKWmEB)^66^ and polygenic-background-control-based least angle regression plus empirical Bayes (pLARmEB)^67^ in mrMLM.GUI in R. For MLM, fixed effects were calculated with a Q (population structure) matrix, and random effects were calculated with a K (Kinship) matrix. The Q + K matrices were both considered in the MLM model. The Q matrix was calculated using the Admixture software package^54^, and the K matrix (the genetic relationship among 149 accessions) was predicted using SPAGeDi software. FaST-LMM uses a linear mixed modeling approach to test SNP association with quantitative traits. For EMMAX, independent SNPs were used to compute the centered relatedness matrix, and the significant P-value between SNPs and phenotypes was calculated. For methods mrMLM, FASTmrMLM, FASTmrEMMA, ISIS EM-BLASSO, pKWmEB and pLARmEB, the methodologies and procedures were processed according to the reports in recent years^62–67^. The result of these analyses can be obtained by using the R network (mrMLM.GUI v3.2, https://cran.r-project.org/web/packages/mrMLM.GUI/index.html).

### Gene identification of associated SNPs

We found the SLAF sequences that the suggestive SNPs located on and used the DNA sequences as queries to conduct BLASTN with the public database. Meanwhile, the SLAF sequences were used to make a conserved domain database analysis using NCBI’s Conserved Domain Database^68^.

## Supplementary information

Supplementary Information

## Data Availability

All of the data generated or analyzed during this study are included in this published article (and its Supplementary Information files).
