# Supplementary material for: Core set construction and association analysis of Pinus massoniana from Guangdong province in southern China using SLAF-seq
Source: Sci Rep. 2019 Sep 11;9:13157. doi: 10.1038/s41598-019-49737-2 (PMC6739479; doi:10.1038/s41598-019-49737-2)
Supplement: Supplementary file 1 — Supplementary Information [file 41598_2019_49737_MOESM1_ESM.docx]

**Core set construction and association analysis of *Pinus massoniana* from Guangdong province in southern China using SLAF-seq**

Qingsong Bai^1,2^ Yanling Cai^1,2^  Boxiang He^1,2^ Wanchuan Liu^3^ Qingyou Pan^3^ Qian Zhang*^1,2^

1. Guangdong Provincial Key Laboratory of Silviculture, Protection and Utilization, Guangdong Academy of Forestry, Guangzhou, 510520, China;

2. Guangdong Academy of Forestry, Guangzhou, 510520, China;

3. Xinyi Forestry Research Institute, Maoming, 525300, China.

* Corresponding author.

E-mail: [zhangq1901@163.com](mailto:zhangq1901@163.com)

**Supplementary Figure S1**

**Population K value selection using Structure and fastStructure**


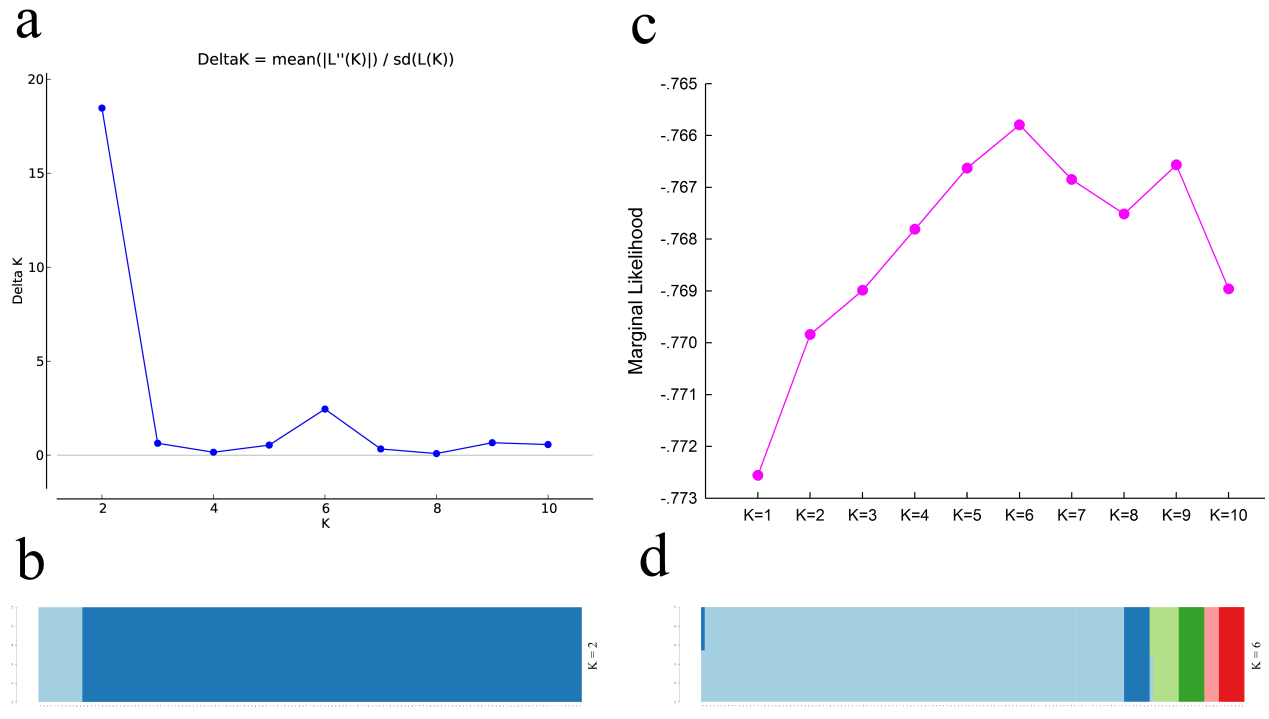


**Supplementary Figure S2**

**Linkage disequilibrium distribution of SNPs**


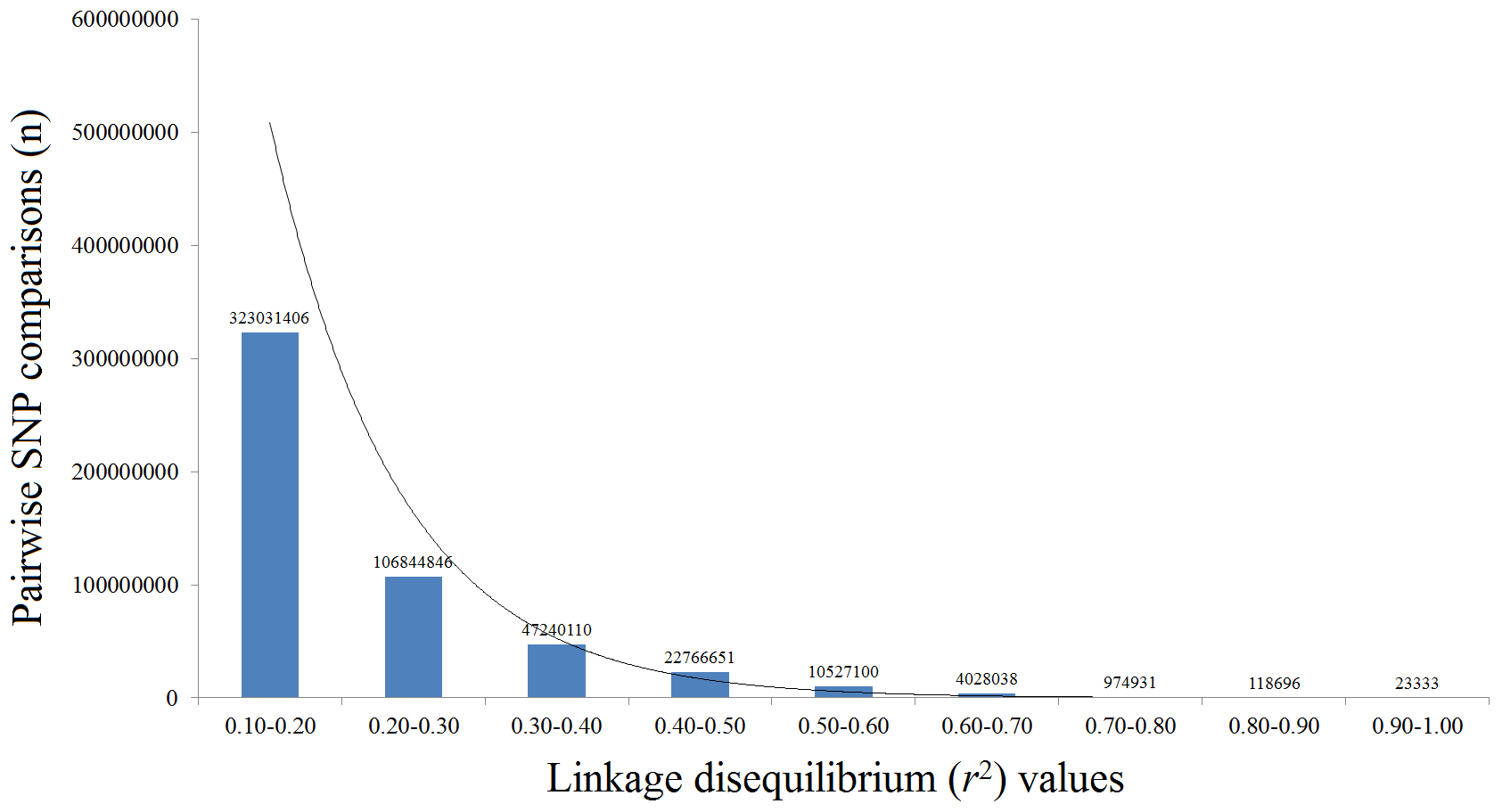


**Supplementary Figure S3**

**The distribution of kinship value among 149 masson pine accessions**


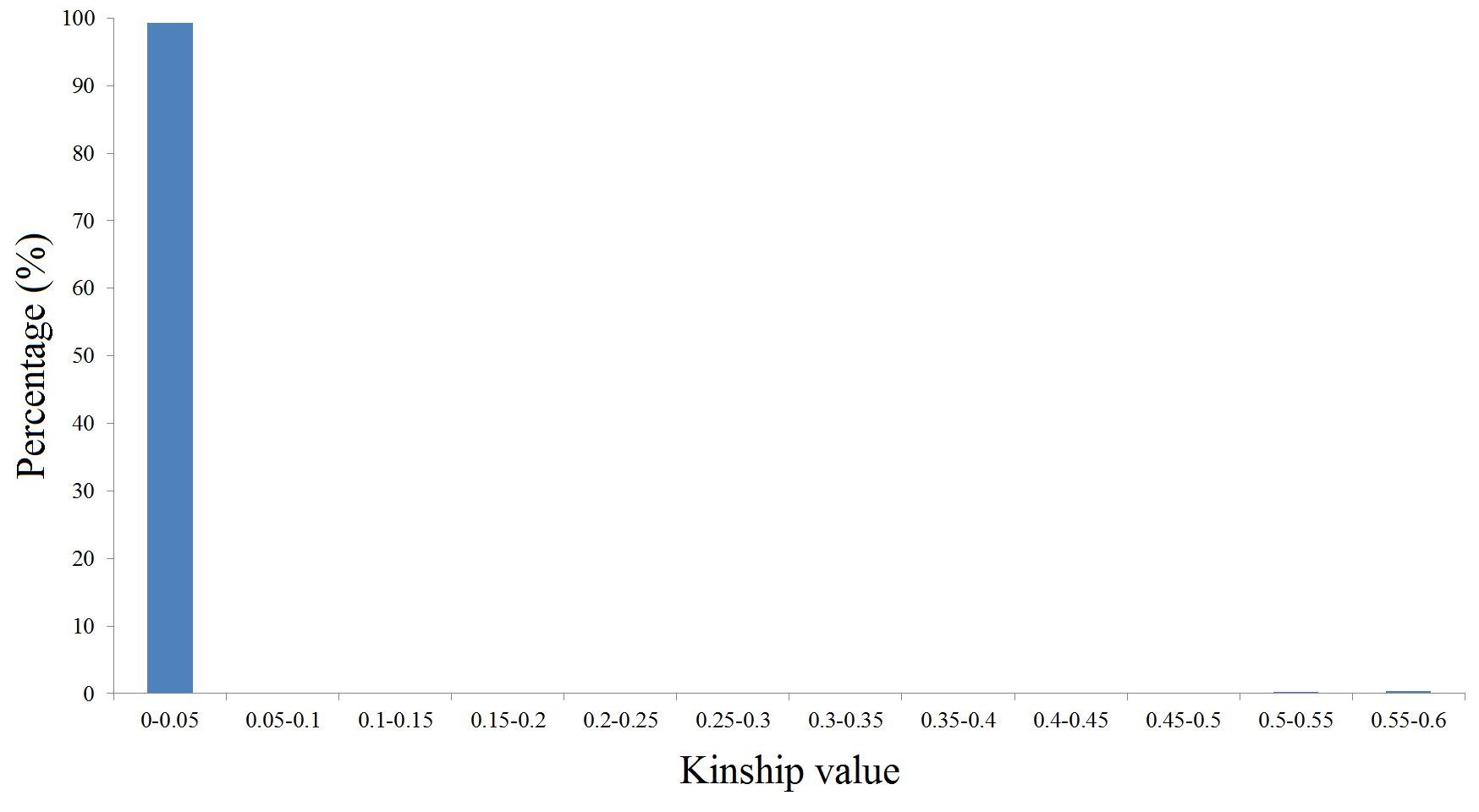


**Supplementary Figure S4**

**The frequency distribution of masson pine traits**


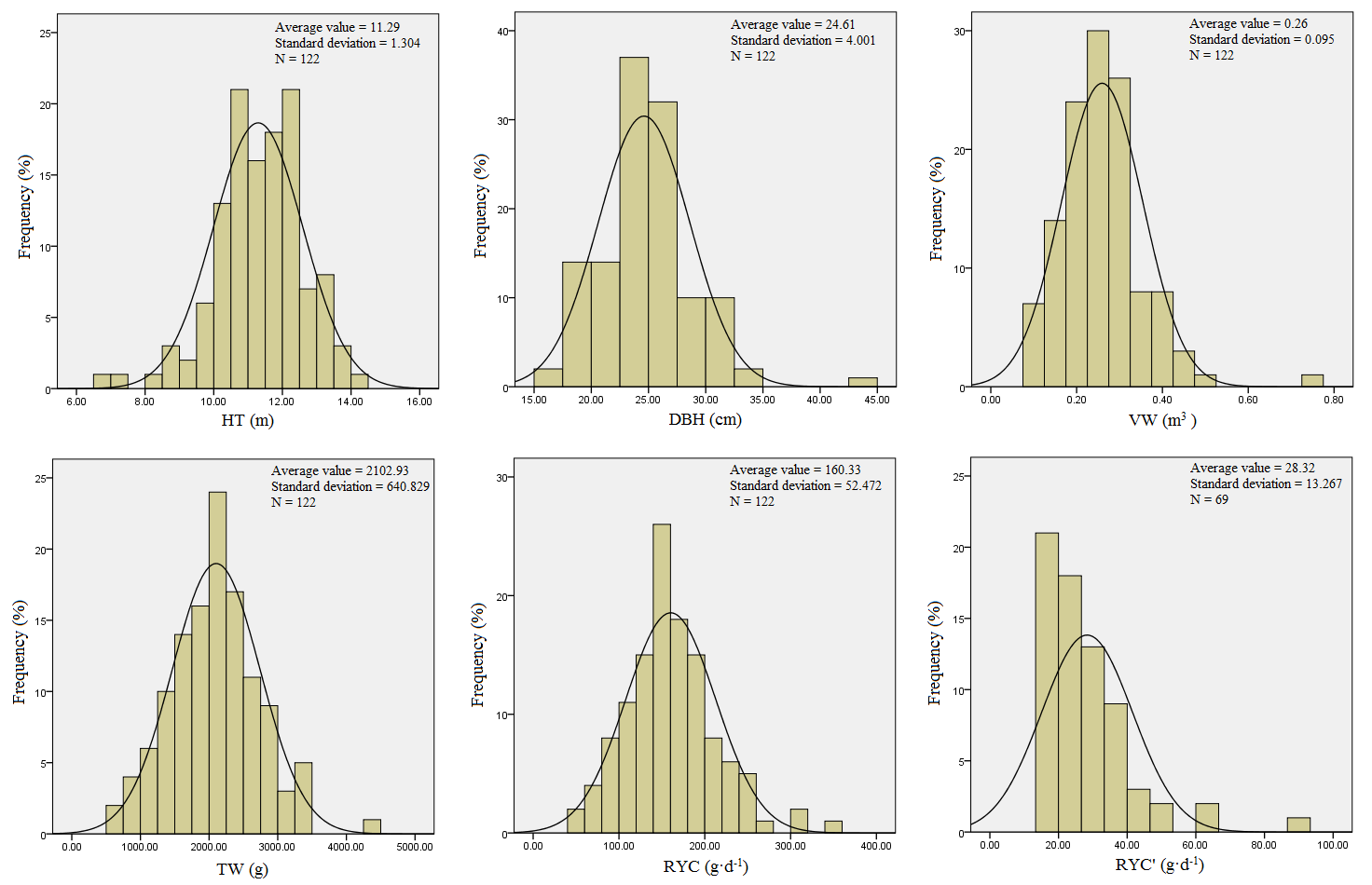


**Supplementary Table S1**

**Location and sequencing statistics of 149 masson pine accessions**

| **Location** | **Sample ID** | **Sequencing ID** | **Total Reads** | **GC Percentage (%)** | **Q30 Percentage (%)** | **Individual number (122 lines)** | **Individual number (69 lines)** |
| --- | --- | --- | --- | --- | --- | --- | --- |
| BL | GW57 | ce | 11690105 | 37.67 | 91.84 | 5 | NA |
|  | GW63 | ck | 9074068 | 37.15 | 92.37 | 2 | NA |
|  | GW64 | cl | 15018394 | 38.53 | 92.6 | 2 | NA |
|  | GW68 | cp | 13265034 | 37.51 | 93.17 | 2 | NA |
|  | GW75 | cw | 11680843 | 37.73 | 92.89 | 7 | NA |
|  | GW76 | cx | 11433663 | 38.55 | 93.32 | 6 | NA |
|  | GW81 | dc | 16273227 | 38.43 | 91.91 | 6 | NA |
|  | GW86 | dh | 9965815 | 38.38 | 92.7 | 4 | NA |
|  | GW99 | du | 8380228 | 37.14 | 93.31 | 2 | NA |
|  | GW119 | eo | 8653716 | 37.09 | 92.55 | 4 | NA |
| CA | GW98 | dt | 7861779 | 37.12 | 92.39 | 5 | NA |
| DQ | GW3 | ac | 12246394 | 37.62 | 92.9 | 2 | 1 |
|  | GW12 | al | 10060686 | 38.02 | 93.62 | 7 | NA |
|  | GW13 | am | 11960775 | 38.14 | 93.09 | 7 | 1 |
|  | GW16 | ap | 18508173 | 37.94 | 93.58 | 5 | 1 |
|  | GW19 | as | 10859029 | 37.22 | 93 | 3 | 1 |
|  | GW39 | bm | 12721038 | 38.05 | 91.93 | 2 | 1 |
|  | GW42 | bp | 9963643 | 37.13 | 93.03 | 2 | 1 |
|  | GW49 | bw | 12276165 | 39.02 | 93.86 | 3 | NA |
|  | GW54 | cb | 16347422 | 38.81 | 93.43 | 5 | 1 |
|  | GW111 | eg | 13828629 | 37.74 | 93.34 | 1 | NA |
|  | GW130 | ez | 14778763 | 37.9 | 93.23 | NA | 1 |
|  | GW143 | fm | 13552378 | 39.13 | 92.52 | 2 | 1 |
| DY | GW5 | ae | 13011665 | 37.06 | 93.25 | 2 | 1 |
|  | GW120 | ep | 8078606 | 37.41 | 92.42 | NA | NA |
|  | GW127 | ew | 8305108 | 38.15 | 91.58 | NA | NA |
|  | GW129 | ey | 14657679 | 38.98 | 92.67 | NA | 1 |
|  | GW133 | fc | 11611058 | 37.91 | 92.66 | NA | 1 |
|  | GW136 | ff | 10518453 | 38.12 | 92.6 | NA | 1 |
|  | GW137 | fg | 17700897 | 39.41 | 92.61 | NA | NA |
|  | GW139 | fi | 11400014 | 37.64 | 93.15 | NA | NA |
| GZ | GW9 | ai | 6625787 | 37.13 | 92.65 | 7 | NA |
|  | GW17 | aq | 12355111 | 36.99 | 92.87 | 3 | 1 |
|  | GW24 | ax | 10583275 | 36.73 | 92.96 | 12 | 1 |
|  | GW29 | bc | 11770538 | 37.56 | 92.99 | 6 | 1 |
|  | GW30 | bd | 6712406 | 37.28 | 92.58 | 3 | 1 |
|  | GW31 | be | 6616287 | 37.17 | 92.21 | 4 | 1 |
|  | GW34 | bh | 14121048 | 38.55 | 93.51 | 5 | 1 |
|  | GW35 | bi | 13753312 | 38.56 | 92.72 | 6 | 1 |
|  | GW45 | bs | 10105131 | 37.5 | 92.91 | 2 | 1 |
|  | GW47 | bu | 8497233 | 37.58 | 93.11 | 6 | 1 |
|  | GW51 | by | 10425612 | 37.52 | 92.27 | 3 | 1 |
|  | GW53 | ca | 13461527 | 38.3 | 92.86 | 2 | 1 |
|  | GW55 | cc | 9736757 | 37.69 | 92.31 | 2 | 1 |
|  | GW79 | da | 10182273 | 37.7 | 93.23 | 11 | NA |
|  | GW105 | ea | 16448654 | 38.27 | 93.01 | 1 | NA |
|  | GW106 | eb | 10856477 | 37.43 | 92.76 | 1 | NA |
|  | GW108 | ed | 12022252 | 40.17 | 93.15 | 2 | NA |
|  | GW110 | ef | 10102635 | 37.48 | 92.25 | 2 | NA |
|  | GW132 | fb | 11761291 | 37.73 | 92.89 | NA | 1 |
| LZ | GW131 | fa | 8529835 | 37.53 | 92.18 | NA | 1 |
|  | GW135 | fe | 11257949 | 37.59 | 91.29 | NA | 1 |
| XY | GW2 | ab | 8885603 | 38.14 | 93.18 | 14 | 1 |
|  | GW4 | ad | 10107673 | 37.43 | 93.2 | 14 | 1 |
|  | GW6 | af | 9832175 | 38.33 | 93.13 | 17 | 1 |
|  | GW7 | ag | 9656289 | 38.41 | 93.26 | 8 | 1 |
|  | GW8 | ah | 11198025 | 37.27 | 92.99 | 14 | NA |
|  | GW10 | aj | 12782123 | 37.66 | 92.74 | 5 | 1 |
|  | GW11 | ak | 10455849 | 38.12 | 93.48 | 2 | 1 |
|  | GW20 | at | 8665360 | 37.16 | 92.69 | 10 | 1 |
|  | GW21 | au | 19549531 | 37.67 | 93.21 | 2 | 1 |
|  | GW22 | av | 15868412 | 38.44 | 92.24 | 2 | 1 |
|  | GW23 | aw | 13368897 | 38.37 | 93.73 | 3 | 1 |
|  | GW26 | az | 16287119 | 38.22 | 93.07 | 13 | 1 |
|  | GW27 | ba | 10703205 | 38.8 | 92.51 | 8 | 1 |
|  | GW28 | bb | 4908642 | 37.61 | 92.42 | 6 | 1 |
|  | GW32 | bf | 10968471 | 37.52 | 92.36 | 8 | 1 |
|  | GW33 | bg | 26491996 | 39.99 | 92.29 | 1 | 1 |
|  | GW36 | bj | 6725970 | 37.58 | 92.39 | 14 | 1 |
|  | GW38 | bl | 15470266 | 38.63 | 93.09 | 7 | 1 |
|  | GW40 | bn | 11954181 | 37.27 | 92.63 | 2 | 1 |
|  | GW41 | bo | 10652313 | 37.71 | 92.79 | 3 | 1 |
|  | GW43 | bq | 13362588 | 38.38 | 91.94 | 6 | 1 |
|  | GW44 | br | 11895645 | 38.09 | 93.35 | 10 | 1 |
|  | GW48 | bv | 10792760 | 37.66 | 92.64 | 5 | 1 |
|  | GW50 | bx | 12431681 | 38.55 | 92.71 | 2 | 1 |
|  | GW56 | cd | 13169302 | 38.33 | 92.88 | 2 | 1 |
|  | GW58 | cf | 18897838 | 37.97 | 93.2 | 10 | NA |
|  | GW60 | ch | 14358152 | 38.55 | 93.65 | 9 | NA |
|  | GW61 | ci | 10376621 | 38.11 | 92.65 | 2 | NA |
|  | GW62 | cj | 10032042 | 37.51 | 93.27 | 2 | NA |
|  | GW65 | cm | 16221910 | 38.97 | 92.63 | 6 | NA |
|  | GW66 | cn | 10004837 | 37.37 | 92.43 | 17 | NA |
|  | GW67 | co | 10395677 | 37.54 | 92.64 | 8 | NA |
|  | GW71 | cs | 12323914 | 38.79 | 93.64 | 4 | NA |
|  | GW72 | ct | 11318760 | 37.54 | 92.64 | 5 | NA |
|  | GW73 | cu | 6458439 | 37.92 | 92.46 | 2 | NA |
|  | GW74 | cv | 10235047 | 38.12 | 92.84 | 4 | NA |
|  | GW77 | cy | 7785175 | 37.25 | 93.25 | 7 | NA |
|  | GW78 | cz | 14346962 | 37.46 | 93.11 | 3 | NA |
|  | GW80 | db | 11635040 | 38.19 | 91.63 | 1 | NA |
|  | GW82 | dd | 15389045 | 37.91 | 92.59 | 3 | NA |
|  | GW83 | de | 14895241 | 37.59 | 92.52 | 8 | NA |
|  | GW85 | dg | 9122458 | 37.43 | 93.34 | 3 | NA |
|  | GW87 | di | 11092251 | 38.03 | 93.15 | 19 | NA |
|  | GW89 | dk | 6868163 | 37.38 | 92.14 | 4 | NA |
|  | GW90 | dl | 10767264 | 37.5 | 92.97 | 1 | NA |
|  | GW92 | dn | 10993807 | 38.33 | 93.69 | 17 | NA |
|  | GW93 | do | 9928472 | 37.32 | 92.48 | 9 | NA |
|  | GW94 | dp | 9732836 | 37.46 | 93.35 | 7 | NA |
|  | GW95 | dq | 15624386 | 37.32 | 93.49 | 8 | NA |
|  | GW97 | ds | 14835671 | 38.38 | 93.43 | 1 | NA |
|  | GW100 | dv | 7404500 | 37.66 | 92.39 | 6 | NA |
|  | GW102 | dx | 13503531 | 37.79 | 92.84 | 8 | NA |
|  | GW103 | dy | 12454968 | 38.73 | 93.27 | 9 | NA |
|  | GW104 | dz | 8663392 | 37.05 | 93.21 | 5 | NA |
|  | GW107 | ec | 9292120 | 39.12 | 93.08 | 7 | NA |
|  | GW109 | ee | 12831215 | 37.72 | 92.48 | 1 | NA |
|  | GW112 | eh | 13105693 | 38.94 | 93.76 | 2 | NA |
|  | GW113 | ei | 12826515 | 39.08 | 92.98 | 2 | NA |
|  | GW114 | ej | 13116354 | 38.09 | 92.51 | 2 | NA |
|  | GW115 | ek | 10436596 | 38.22 | 92.39 | 2 | NA |
|  | GW116 | el | 9674892 | 38.43 | 91.66 | 1 | NA |
|  | GW117 | em | 13168057 | 37.56 | 93.05 | 1 | NA |
|  | GW118 | en | 10508384 | 37.88 | 92.95 | 1 | NA |
|  | GW122 | er | 11818247 | 38.96 | 92.87 | NA | 1 |
|  | GW141 | fk | 7502946 | 37.38 | 91.19 | NA | NA |
|  | GW142 | fl | 10230013 | 38.69 | 92.33 | 3 | NA |
|  | GW146 | fp | 12666163 | 37.18 | 93.45 | NA | NA |
| YD | GW59 | cg | 12246548 | 37.63 | 92.52 | 8 | NA |
|  | GW88 | dj | 8269909 | 36.91 | 93.13 | 1 | NA |
|  | GW101 | dw | 5941835 | 37.41 | 92.81 | 6 | NA |
| YN | GW1 | aa | 16553301 | 38.49 | 92.55 | 2 | 1 |
|  | GW14 | an | 9429174 | 37.09 | 92.37 | 2 | 1 |
|  | GW15 | ao | 17248743 | 37.28 | 93.13 | 5 | 1 |
|  | GW18 | ar | 16236918 | 38.59 | 92.89 | 2 | 1 |
|  | GW25 | ay | 12933137 | 37.3 | 93.08 | 4 | 1 |
|  | GW37 | bk | 19507739 | 38.94 | 92.53 | 5 | 1 |
|  | GW46 | bt | 8874944 | 37.58 | 92.93 | 2 | 1 |
|  | GW52 | bz | 22264039 | 38.53 | 91.93 | 3 | 1 |
|  | GW69 | cq | 17622189 | 38.3 | 92.45 | 1 | NA |
|  | GW70 | cr | 9832158 | 37.59 | 93.33 | 2 | NA |
|  | GW84 | df | 11848177 | 37.76 | 92.25 | 3 | NA |
|  | GW91 | dm | 9860993 | 38.3 | 92.32 | 1 | NA |
|  | GW96 | dr | 12949835 | 38.35 | 92.64 | 3 | NA |
|  | GW121 | eq | 10363223 | 38.74 | 92.33 | NA | 1 |
|  | GW123 | es | 14844598 | 38.71 | 92.93 | NA | 1 |
|  | GW124 | et | 9903664 | 37.54 | 92.37 | NA | NA |
|  | GW125 | eu | 8604364 | 37.45 | 92.93 | NA | 1 |
|  | GW126 | ev | 10165740 | 38.69 | 92.39 | NA | NA |
|  | GW128 | ex | 15257318 | 38.35 | 92.47 | NA | 1 |
|  | GW134 | fd | 9939457 | 38.63 | 93.31 | NA | NA |
|  | GW138 | fh | 12255101 | 37.45 | 91.51 | NA | NA |
|  | GW140 | fj | 10930858 | 37.65 | 92.43 | NA | 1 |
|  | GW144 | fn | 11702132 | 38.38 | 93.48 | NA | 1 |
|  | GW145 | fo | 12518621 | 37.58 | 93.37 | NA | 1 |
|  | GW147 | fq | 7884517 | 37.58 | 92.06 | 3 | NA |
|  | GW148 | fr | 8760718 | 37.44 | 93 | NA | 1 |
|  | GW149 | fs | 13818690 | 38.7 | 92.68 | NA | NA |

**Supplementary Table S2**

**The information of the identified SNPs among 149 masson pine accessions**

| **Sample ID** | **SLAF number** | **Total depth** | **Average depth** | **Total SNP** | **SNP number** | **Hetloci ratio(%)** |
| --- | --- | --- | --- | --- | --- | --- |
| GW1 | 706526 | 13521876 | 19.1385 | 2774976 | 1302178 | 9.49 |
| GW2 | 611810 | 8051269 | 13.1598 | 2774976 | 1013394 | 9.14 |
| GW3 | 599741 | 11249456 | 18.7572 | 2774976 | 975767 | 8.74 |
| GW4 | 586985 | 9255877 | 15.7685 | 2774976 | 923654 | 8.37 |
| GW5 | 592658 | 12021036 | 20.2833 | 2774976 | 936210 | 7.92 |
| GW6 | 629552 | 8972851 | 14.2528 | 2774976 | 1055135 | 8.79 |
| GW7 | 625998 | 8772620 | 14.0138 | 2774976 | 1048136 | 9.1 |
| GW8 | 581446 | 10233343 | 17.5998 | 2774976 | 918801 | 8.18 |
| GW9 | 527741 | 6048797 | 11.4617 | 2774976 | 808047 | 7.1 |
| GW10 | 610441 | 11747142 | 19.2437 | 2774976 | 1013970 | 8.62 |
| GW11 | 618291 | 9670688 | 15.641 | 2774976 | 1021921 | 8.93 |
| GW12 | 612661 | 9267941 | 15.1274 | 2774976 | 1005816 | 8.85 |
| GW13 | 595316 | 10959251 | 18.4091 | 2774976 | 961344 | 8.56 |
| GW14 | 549723 | 8524405 | 15.5067 | 2774976 | 855067 | 7.61 |
| GW15 | 628777 | 15813363 | 25.1494 | 2774976 | 1006926 | 8.82 |
| GW16 | 665257 | 17056745 | 25.6393 | 2774976 | 1163041 | 10.02 |
| GW17 | 604163 | 11247199 | 18.6162 | 2774976 | 978563 | 8.81 |
| GW18 | 640105 | 14687841 | 22.946 | 2774976 | 1071468 | 9.7 |
| GW19 | 564341 | 9966662 | 17.6607 | 2774976 | 864784 | 8 |
| GW20 | 571814 | 7853239 | 13.7339 | 2774976 | 898060 | 7.96 |
| GW21 | 642746 | 17961582 | 27.9451 | 2774976 | 1052329 | 9.34 |
| GW22 | 654829 | 14027799 | 21.4221 | 2774976 | 1135686 | 9.26 |
| GW23 | 656178 | 12199386 | 18.5916 | 2774976 | 1120212 | 9.79 |
| GW24 | 586946 | 9694171 | 16.5163 | 2774976 | 935943 | 8.74 |
| GW25 | 624497 | 11610288 | 18.5914 | 2774976 | 992785 | 8.58 |
| GW26 | 651436 | 14681508 | 22.5371 | 2774976 | 1150147 | 9.21 |
| GW27 | 637960 | 9598820 | 15.0461 | 2774976 | 1099285 | 9.11 |
| GW28 | 530665 | 4420282 | 8.3297 | 2774976 | 839100 | 6.65 |
| GW29 | 603937 | 10736898 | 17.7782 | 2774976 | 989716 | 8.65 |
| GW30 | 543292 | 6024291 | 11.0885 | 2774976 | 835276 | 7.42 |
| GW31 | 517555 | 6004553 | 11.6018 | 2774976 | 791468 | 6.98 |
| GW32 | 578766 | 9975463 | 17.2357 | 2774976 | 915488 | 8.17 |
| GW33 | 702743 | 23515266 | 33.4621 | 2774976 | 1308510 | 10.55 |
| GW34 | 649980 | 12966209 | 19.9486 | 2774976 | 1131415 | 9.59 |
| GW35 | 643239 | 12560447 | 19.5269 | 2774976 | 1110044 | 9.24 |
| GW36 | 566667 | 6001731 | 10.5913 | 2774976 | 950300 | 7.31 |
| GW37 | 672056 | 17654230 | 26.269 | 2774976 | 1206488 | 10.3 |
| GW38 | 651306 | 14147365 | 21.7215 | 2774976 | 1122248 | 9.48 |
| GW39 | 633206 | 11424721 | 18.0427 | 2774976 | 1054878 | 9.21 |
| GW40 | 567252 | 10834517 | 19.1 | 2774976 | 892750 | 7.79 |
| GW41 | 594353 | 9750374 | 16.405 | 2774976 | 961200 | 8.55 |
| GW42 | 563118 | 9157453 | 16.262 | 2774976 | 886087 | 7.88 |
| GW43 | 638247 | 12085754 | 18.9359 | 2774976 | 1081588 | 9.32 |
| GW44 | 616288 | 10932032 | 17.7385 | 2774976 | 1026655 | 8.87 |
| GW45 | 562211 | 9272093 | 16.4922 | 2774976 | 879338 | 7.76 |
| GW46 | 569005 | 8096818 | 14.2298 | 2774976 | 880343 | 7.49 |
| GW47 | 556279 | 7810394 | 14.0404 | 2774976 | 880394 | 7.22 |
| GW48 | 583769 | 9860388 | 16.8909 | 2774976 | 936206 | 8.5 |
| GW49 | 657467 | 11238332 | 17.0934 | 2774976 | 1144246 | 9.39 |
| GW50 | 628946 | 11141988 | 17.7153 | 2774976 | 1066686 | 9.29 |
| GW51 | 582237 | 9398296 | 16.1417 | 2774976 | 928122 | 8.09 |
| GW52 | 664220 | 19981234 | 30.0823 | 2774976 | 1169069 | 9.85 |
| GW53 | 641553 | 12348894 | 19.2484 | 2774976 | 1077951 | 9.19 |
| GW54 | 669600 | 14983800 | 22.3772 | 2774976 | 1172479 | 9.65 |
| GW55 | 571709 | 8684355 | 15.1902 | 2774976 | 909768 | 7.81 |
| GW56 | 643167 | 11789443 | 18.3303 | 2774976 | 1099624 | 9.1 |
| GW57 | 624823 | 10337320 | 16.5444 | 2774976 | 1025804 | 8.52 |
| GW58 | 634143 | 17461931 | 27.5363 | 2774976 | 1050550 | 9.34 |
| GW59 | 592591 | 11245329 | 18.9765 | 2774976 | 954873 | 8.24 |
| GW60 | 655066 | 13142868 | 20.0634 | 2774976 | 1131609 | 9.69 |
| GW61 | 612691 | 9377204 | 15.3049 | 2774976 | 994819 | 8.75 |
| GW62 | 611710 | 9158095 | 14.9713 | 2774976 | 952307 | 8.25 |
| GW63 | 587061 | 8049509 | 13.7115 | 2774976 | 889614 | 7.63 |
| GW64 | 652968 | 13569366 | 20.7811 | 2774976 | 1109980 | 9.7 |
| GW65 | 656683 | 14574012 | 22.1934 | 2774976 | 1140669 | 9.92 |
| GW66 | 589057 | 9007461 | 15.2913 | 2774976 | 935295 | 8.12 |
| GW67 | 644799 | 9171198 | 14.2233 | 2774976 | 985036 | 8.53 |
| GW68 | 644196 | 12002495 | 18.6317 | 2774976 | 1042483 | 8.57 |
| GW69 | 675206 | 15708025 | 23.264 | 2774976 | 1167237 | 9.51 |
| GW70 | 704177 | 8519054 | 12.0979 | 2774976 | 962092 | 7.36 |
| GW71 | 622171 | 11354194 | 18.2493 | 2774976 | 1032496 | 9.36 |
| GW72 | 608531 | 10192753 | 16.7498 | 2774976 | 982019 | 8.7 |
| GW73 | 569745 | 5754705 | 10.1005 | 2774976 | 911657 | 7.97 |
| GW74 | 623177 | 9192326 | 14.7507 | 2774976 | 1040122 | 8.77 |
| GW75 | 620019 | 10681940 | 17.2284 | 2774976 | 1015849 | 9.19 |
| GW76 | 645847 | 10268647 | 15.8995 | 2774976 | 1108294 | 9.29 |
| GW77 | 588090 | 7059048 | 12.0033 | 2774976 | 943062 | 8.06 |
| GW78 | 669004 | 12719839 | 19.0131 | 2774976 | 1067994 | 9.58 |
| GW79 | 604548 | 9265389 | 15.3261 | 2774976 | 998426 | 8.36 |
| GW80 | 623752 | 10341265 | 16.5791 | 2774976 | 1042701 | 9.21 |
| GW81 | 673128 | 14370338 | 21.3486 | 2774976 | 1142807 | 10.21 |
| GW82 | 679002 | 13706380 | 20.1861 | 2774976 | 1079199 | 9.33 |
| GW83 | 653262 | 13258988 | 20.2966 | 2774976 | 1067658 | 9.67 |
| GW84 | 663325 | 10273738 | 15.4882 | 2774976 | 1034623 | 8.1 |
| GW85 | 602368 | 8322406 | 13.8161 | 2774976 | 982258 | 8.7 |
| GW86 | 630132 | 8858539 | 14.0582 | 2774976 | 1080503 | 9.35 |
| GW87 | 640323 | 9619515 | 15.0229 | 2774976 | 1073945 | 8.95 |
| GW88 | 567788 | 7522902 | 13.2495 | 2774976 | 879948 | 7.32 |
| GW89 | 582827 | 6021196 | 10.331 | 2774976 | 908287 | 8.06 |
| GW90 | 620348 | 9701928 | 15.6395 | 2774976 | 1007008 | 8.55 |
| GW91 | 623199 | 8710175 | 13.9766 | 2774976 | 1051229 | 8.86 |
| GW92 | 641776 | 9925110 | 15.4651 | 2774976 | 1098072 | 9.12 |
| GW93 | 621018 | 8724068 | 14.048 | 2774976 | 973305 | 8.61 |
| GW94 | 629046 | 8655284 | 13.7594 | 2774976 | 989530 | 8.7 |
| GW95 | 652434 | 14271894 | 21.8748 | 2774976 | 1058801 | 9.14 |
| GW96 | 650732 | 11333984 | 17.4173 | 2774976 | 1114704 | 9.37 |
| GW97 | 666402 | 13280641 | 19.9289 | 2774976 | 1165025 | 9.59 |
| GW98 | 571233 | 7038580 | 12.3217 | 2774976 | 906809 | 8.03 |
| GW99 | 577963 | 7611957 | 13.1703 | 2774976 | 895429 | 7.65 |
| GW100 | 582172 | 6606067 | 11.3473 | 2774976 | 926734 | 8.16 |
| GW101 | 552661 | 5417641 | 9.8028 | 2774976 | 873253 | 7.24 |
| GW102 | 634826 | 12291567 | 19.3621 | 2774976 | 1055075 | 9.47 |
| GW103 | 641760 | 11395003 | 17.7559 | 2774976 | 1088083 | 9.27 |
| GW104 | 576482 | 7973855 | 13.8319 | 2774976 | 911537 | 8.06 |
| GW105 | 630157 | 15168086 | 24.0703 | 2774976 | 1054334 | 9.39 |
| GW106 | 609919 | 9916880 | 16.2593 | 2774976 | 984808 | 8.99 |
| GW107 | 632335 | 8428680 | 13.3295 | 2774976 | 1080007 | 9.29 |
| GW108 | 660833 | 10666503 | 16.141 | 2774976 | 1169763 | 10.07 |
| GW109 | 624968 | 11500597 | 18.4019 | 2774976 | 1014162 | 8.66 |
| GW110 | 604949 | 9108498 | 15.0566 | 2774976 | 975457 | 9.01 |
| GW111 | 637604 | 12684426 | 19.8939 | 2774976 | 1088697 | 9.21 |
| GW112 | 662564 | 12016159 | 18.1358 | 2774976 | 1145658 | 10.16 |
| GW113 | 654207 | 11571157 | 17.6873 | 2774976 | 1150552 | 9.76 |
| GW114 | 633756 | 11907621 | 18.789 | 2774976 | 1056656 | 9.4 |
| GW115 | 614007 | 9261797 | 15.0842 | 2774976 | 1015148 | 8.9 |
| GW116 | 612656 | 8525798 | 13.9161 | 2774976 | 1001866 | 8.8 |
| GW117 | 627611 | 12086367 | 19.2577 | 2774976 | 1021293 | 9.18 |
| GW118 | 625826 | 9545707 | 15.253 | 2774976 | 1034912 | 8.98 |
| GW119 | 578315 | 7829288 | 13.5381 | 2774976 | 921337 | 8.42 |
| GW120 | 591211 | 7179976 | 12.1445 | 2774976 | 924861 | 7.52 |
| GW121 | 626167 | 9270295 | 14.8048 | 2774976 | 1062089 | 9.09 |
| GW122 | 671262 | 10582893 | 15.7657 | 2774976 | 1185324 | 9.98 |
| GW123 | 673995 | 13051648 | 19.3646 | 2774976 | 1199610 | 9.66 |
| GW124 | 598639 | 8922645 | 14.9049 | 2774976 | 964660 | 8.74 |
| GW125 | 597130 | 7709738 | 12.9113 | 2774976 | 957019 | 8.33 |
| GW126 | 640887 | 8894654 | 13.8787 | 2774976 | 1134104 | 9.26 |
| GW127 | 611056 | 7373017 | 12.066 | 2774976 | 1005566 | 8.97 |
| GW128 | 655849 | 13635696 | 20.7909 | 2774976 | 1119985 | 9.9 |
| GW129 | 667227 | 13077558 | 19.5999 | 2774976 | 1172433 | 9.48 |
| GW130 | 652068 | 13421270 | 20.5826 | 2774976 | 1083867 | 9.1 |
| GW131 | 629434 | 7289588 | 11.5812 | 2774976 | 964441 | 7.96 |
| GW132 | 658594 | 10410247 | 15.8068 | 2774976 | 1040370 | 8.89 |
| GW133 | 635287 | 10501691 | 16.5306 | 2774976 | 1060096 | 9.11 |
| GW134 | 643720 | 8872133 | 13.7826 | 2774976 | 1092053 | 9.27 |
| GW135 | 611910 | 9774617 | 15.9739 | 2774976 | 1003152 | 8.47 |
| GW136 | 633923 | 9139831 | 14.4179 | 2774976 | 1024256 | 8.8 |
| GW137 | 684192 | 15433751 | 22.5576 | 2774976 | 1290584 | 10.86 |
| GW138 | 599743 | 10986791 | 18.3192 | 2774976 | 961036 | 8.72 |
| GW139 | 650681 | 10164810 | 15.6218 | 2774976 | 1008555 | 8.49 |
| GW140 | 628877 | 9863985 | 15.6851 | 2774976 | 1018284 | 8.47 |
| GW141 | 592058 | 6436043 | 10.8706 | 2774976 | 913715 | 7.84 |
| GW142 | 638419 | 8871596 | 13.8962 | 2774976 | 1032792 | 9.09 |
| GW143 | 670112 | 11995822 | 17.9012 | 2774976 | 1174804 | 10.08 |
| GW144 | 637439 | 10706944 | 16.7968 | 2774976 | 1070435 | 9.72 |
| GW145 | 618794 | 11453702 | 18.5097 | 2774976 | 1007541 | 8.84 |
| GW146 | 618804 | 11612594 | 18.7662 | 2774976 | 996683 | 8.78 |
| GW147 | 596998 | 6897645 | 11.5539 | 2774976 | 933995 | 7.93 |
| GW148 | 591265 | 7976943 | 13.4913 | 2774976 | 951500 | 8.28 |
| GW149 | 660255 | 12347500 | 18.7011 | 2774976 | 1154949 | 9.57 |

**Supplementary Table S3**

**Associated SNPs according to P-value using mrMLM and FASTmrMLM**

| **Trait** | **Marker** | **Position** | **Alleles** | **P-value** |
| --- | --- | --- | --- | --- |
| HT | Marker167996 | 196 | A/G | 3.01E-12 |
|  | Marker463568 | 29 | G/T | 3.28E-10 |
|  | Marker137969 | 101 | A/G | 2.96E-09 |
|  | Marker3638917 | 217 | T/C | 4.51E-09 |
|  | Marker235063 | 223 | C/G | 8.84E-09 |
|  | Marker103914 | 110 | G/A | 1.51E-08 |
|  | Marker169405 | 106 | C/A | 1.51E-08 |
|  | Marker148450 | 85 | G/A | 4.98E-08 |
|  | Marker150202 | 256 | C/T | 4.98E-08 |
|  | Marker169667 | 254 | C/T | 1.3E-07 |
|  | Marker4625809 | 68 | G/A | 1.35E-07 |
|  | Marker163256 | 68 | C/A | 1.89E-07 |
| DBH | Marker279986 | 82 | C/T | 3.9E-08 |
|  | Marker308123 | 258 | T/C | 7.56E-08 |
|  | Marker6395263 | 73 | A/G | 8.54E-08 |
|  | Marker337562 | 207 | G/A | 1.95E-07 |
| RW | Marker342635 | 169 | G/A | 5.53E-09 |
|  | Marker394022 | 171 | T/C | 6.86E-09 |
|  | Marker193988 | 242 | G/T | 7.69E-09 |
|  | Marker502947 | 19 | A/G | 1.32E-08 |
|  | Marker205989 | 112 | C/T | 3.16E-08 |
|  | Marker513429 | 34 | G/A | 7.93E-08 |
|  | Marker153941 | 28 | C/T | 9.25E-08 |
|  | Marker153941 | 80 | C/T | 9.25E-08 |
|  | Marker219564 | 252 | C/T | 1.14E-07 |
|  | Marker248321 | 102 | T/A | 1.49E-07 |
|  | Marker104674 | 196 | G/A | 1.68E-07 |
|  | Marker6913393 | 45 | G/T | 1.79E-07 |
|  | Marker6913393 | 205 | G/A | 1.79E-07 |
|  | Marker262216 | 4 | C/T | 1.88E-07 |
| RYC | Marker394022 | 171 | T/C | 2.22E-16 |
|  | Marker342635 | 169 | G/A | 6.38E-13 |
|  | Marker513429 | 34 | G/A | 1.58E-12 |
|  | Marker284812 | 166 | G/A | 1.69E-12 |
|  | Marker219564 | 252 | C/T | 2.71E-12 |
|  | Marker205989 | 112 | C/T | 3.83E-12 |
|  | Marker109152 | 211 | G/A | 2.06E-11 |
|  | Marker144539 | 14 | G/T | 2.06E-11 |
|  | Marker144539 | 42 | G/A | 2.06E-11 |
|  | Marker315045 | 225 | C/A | 2.06E-11 |
|  | Marker318183 | 243 | T/C | 2.67E-11 |
|  | Marker6913393 | 45 | G/T | 3.71E-11 |
|  | Marker6913393 | 205 | G/A | 3.71E-11 |
|  | Marker104674 | 196 | G/A | 4.26E-11 |
|  | Marker155413 | 36 | C/T | 4.42E-11 |
|  | Marker291839 | 220 | C/T | 4.78E-11 |
|  | Marker291839 | 221 | G/C | 4.78E-11 |
|  | Marker262216 | 4 | C/T | 6.5E-11 |
|  | Marker178718 | 217 | T/C | 7.29E-11 |
|  | Marker1271871 | 11 | C/A | 7.29E-11 |
|  | Marker1271871 | 79 | C/T | 7.29E-11 |
|  | Marker1271871 | 87 | G/A | 7.29E-11 |
|  | Marker1271871 | 106 | C/A | 7.29E-11 |
|  | Marker1271871 | 150 | T/A | 7.29E-11 |
|  | Marker1271871 | 221 | G/A | 7.29E-11 |
|  | Marker1271871 | 236 | C/T | 7.29E-11 |
|  | Marker1271871 | 243 | A/C | 7.29E-11 |
|  | Marker1271871 | 250 | A/T | 7.29E-11 |
|  | Marker243116 | 27 | G/T | 7.8E-11 |
|  | Marker281457 | 164 | A/T | 7.8E-11 |
|  | Marker281457 | 189 | G/T | 7.8E-11 |
|  | Marker248321 | 102 | T/A | 1.44E-10 |
|  | Marker144300 | 159 | C/G | 2.09E-10 |
|  | Marker197273 | 84 | T/C | 3.41E-10 |
|  | Marker153941 | 28 | C/T | 3.9E-10 |
|  | Marker153941 | 80 | C/T | 3.9E-10 |
|  | Marker178771 | 62 | C/T | 5.09E-10 |
|  | Marker502947 | 19 | A/G | 5.35E-10 |
|  | Marker99506 | 175 | G/A | 7.26E-10 |
|  | Marker116962 | 228 | C/A | 7.26E-10 |
|  | Marker99845 | 38 | A/G | 7.47E-10 |
|  | Marker106383 | 192 | C/G | 7.47E-10 |
|  | Marker106383 | 246 | C/T | 7.47E-10 |
|  | Marker238561 | 23 | T/A | 8.13E-10 |
|  | Marker217315 | 18 | G/A | 9.66E-10 |
|  | Marker257361 | 102 | T/A | 1.08E-09 |
|  | Marker227533 | 207 | G/T | 1.32E-09 |
|  | Marker231632 | 211 | A/T | 1.88E-09 |
|  | Marker212473 | 249 | G/T | 3.64E-09 |
|  | Marker106263 | 187 | C/T | 4.03E-09 |
|  | Marker206064 | 232 | G/A | 4.03E-09 |
|  | Marker227691 | 29 | C/G | 4.09E-09 |
|  | Marker217339 | 12 | A/G | 4.62E-09 |
|  | Marker487493 | 24 | G/A | 4.79E-09 |
|  | Marker353032 | 216 | C/T | 5.33E-09 |
|  | Marker616899 | 20 | G/A | 6.6E-09 |
|  | Marker121267 | 104 | G/A | 7.05E-09 |
|  | Marker144043 | 204 | G/A | 7.22E-09 |
|  | Marker144043 | 211 | C/T | 7.22E-09 |
|  | Marker144043 | 230 | A/G | 7.22E-09 |
|  | Marker169205 | 179 | G/A | 8.34E-09 |
|  | Marker243174 | 57 | C/T | 8.34E-09 |
|  | Marker184861 | 77 | C/T | 9.04E-09 |
|  | Marker115413 | 114 | A/C | 9.57E-09 |
|  | Marker115413 | 115 | A/C | 9.57E-09 |
|  | Marker151560 | 97 | T/C | 9.57E-09 |
|  | Marker154657 | 77 | C/T | 9.57E-09 |
|  | Marker199510 | 189 | G/A | 9.57E-09 |
|  | Marker212909 | 157 | C/A | 9.57E-09 |
|  | Marker278201 | 171 | T/C | 1.12E-08 |
|  | Marker6111791 | 198 | T/A | 1.28E-08 |
|  | Marker232253 | 40 | C/T | 1.67E-08 |
|  | Marker164211 | 97 | C/G | 1.78E-08 |
|  | Marker164211 | 156 | T/G | 1.78E-08 |
|  | Marker113907 | 44 | T/C | 1.79E-08 |
|  | Marker375391 | 28 | G/C | 1.8E-08 |
|  | Marker252619 | 116 | T/A | 2.2E-08 |
|  | Marker216699 | 80 | A/C | 2.85E-08 |
|  | Marker317614 | 219 | A/G | 2.87E-08 |
|  | Marker317614 | 224 | A/G | 2.87E-08 |
|  | Marker227082 | 63 | T/C | 3.01E-08 |
|  | Marker296909 | 158 | C/T | 3.08E-08 |
|  | Marker478728 | 160 | C/T | 3.5E-08 |
|  | Marker1182500 | 95 | G/A | 3.56E-08 |
|  | Marker521345 | 251 | C/A | 3.67E-08 |
|  | Marker6846919 | 248 | C/T | 3.68E-08 |
|  | Marker232253 | 107 | C/T | 3.71E-08 |
|  | Marker193988 | 242 | G/T | 3.83E-08 |
|  | Marker405504 | 41 | T/C | 4.19E-08 |
|  | Marker405504 | 42 | A/G | 4.19E-08 |
|  | Marker405504 | 63 | C/A | 4.19E-08 |
|  | Marker405504 | 110 | T/C | 4.19E-08 |
|  | Marker405504 | 113 | T/C | 4.19E-08 |
|  | Marker405504 | 142 | A/G | 4.19E-08 |
|  | Marker405504 | 246 | A/C | 4.19E-08 |
|  | Marker405504 | 258 | T/G | 4.19E-08 |
|  | Marker276259 | 253 | C/A | 4.8E-08 |
|  | Marker596864 | 48 | C/A | 5.23E-08 |
|  | Marker320005 | 180 | A/G | 5.31E-08 |
|  | Marker147954 | 173 | T/C | 6.7E-08 |
|  | Marker213683 | 149 | C/T | 6.94E-08 |
|  | Marker297563 | 163 | A/G | 7.21E-08 |
|  | Marker297563 | 250 | A/G | 7.21E-08 |
|  | Marker111495 | 29 | G/A | 7.68E-08 |
|  | Marker5593053 | 258 | C/T | 7.68E-08 |
|  | Marker292651 | 106 | G/A | 8.01E-08 |
|  | Marker188501 | 40 | T/A | 8.09E-08 |
|  | Marker264845 | 184 | G/A | 8.56E-08 |
|  | Marker174551 | 8 | T/C | 9.34E-08 |
|  | Marker211906 | 242 | G/A | 1.01E-07 |
|  | Marker193030 | 44 | G/A | 1.16E-07 |
|  | Marker126159 | 255 | T/C | 1.21E-07 |
|  | Marker275987 | 207 | G/T | 1.32E-07 |
|  | Marker273939 | 114 | C/T | 1.36E-07 |
|  | Marker273939 | 203 | T/C | 1.36E-07 |
|  | Marker1146383 | 110 | T/C | 1.42E-07 |
|  | Marker1146383 | 225 | A/G | 1.42E-07 |
|  | Marker1146383 | 254 | A/G | 1.42E-07 |
|  | Marker645333 | 188 | A/T | 1.44E-07 |
|  | Marker268196 | 151 | T/C | 1.54E-07 |
|  | Marker268196 | 230 | G/T | 1.54E-07 |
|  | Marker312160 | 193 | G/A | 1.56E-07 |
|  | Marker115068 | 160 | C/T | 1.62E-07 |
|  | Marker257582 | 183 | C/T | 1.74E-07 |
|  | Marker103777 | 93 | C/T | 1.75E-07 |
|  | Marker195484 | 215 | T/C | 1.75E-07 |
|  | Marker253293 | 237 | T/A | 1.75E-07 |
|  | Marker373435 | 234 | A/G | 1.78E-07 |
|  | Marker330391 | 13 | G/T | 1.8E-07 |
|  | Marker533819 | 243 | G/A | 1.86E-07 |
|  | Marker101638 | 245 | G/A | 2E-07 |
|  | Marker159468 | 174 | G/C | 2.03E-07 |
|  | Marker225158 | 181 | A/G | 2.03E-07 |
|  | Marker225158 | 194 | C/T | 2.03E-07 |
|  | Marker279314 | 73 | A/T | 2.03E-07 |
|  | Marker362454 | 240 | C/A | 2.03E-07 |

**Supplementary Table S4**

**Associated SNPs according to LOD values**

| **Trait** | **Marker** | **Position** | **Method** | **LOD** | ***r*^2^ (%)** |
| --- | --- | --- | --- | --- | --- |
| RYC’ | Marker279561 | 86 | mrMLM | 3.1421 | 13.9289 |
|  |  |  | FASTmrMLM | 8.3673 | 23.5109 |
|  |  |  | ISIS EM-BLASSO | 6.1555 | 19.5775 |
|  | Marker357110 | 151 | mrMLM | 5.2969 | 21.5762 |
|  |  |  | FASTmrMLM | 7.3526 | 23.0379 |
|  |  |  | ISIS EM-BLASSO | 5.3265 | 19.3797 |
|  | Marker397927 | 193 | FASTmrMLM | 7.0811 | 12.4827 |
|  |  |  | ISIS EM-BLASSO | 8.4222 | 18.3813 |
|  | Marker424151 | 181 | FASTmrEMMA | 5.4012 | 7.2455 |
|  |  |  | FASTmrMLM | 3.1332 | 1.6266 |
|  | Marker586054 | 168 | mrMLM | 3.7561 | 7.8213 |
|  |  |  | FASTmrMLM | 5.8869 | 9.0131 |
|  |  |  | ISIS EM-BLASSO | 5.1802 | 8.615 |
| VW | Marker5223193 | 162 | FASTmrMLM | 8.6788 | 0.3208 |
|  |  |  | ISIS EM-BLASSO | 5.4032 | 0.9779 |
|  | Marker289071 | 186 | FASTmrMLM | 20.7882 | 1.3581 |
|  |  |  | ISIS EM-BLASSO | 7.2959 | 29.238 |
|  | Marker331316 | 41 | FASTmrMLM | 21.9272 | 0.8568 |
|  |  |  | ISIS EM-BLASSO | 3.1155 | 18.2944 |
|  | Marker490183 | 52 | mrMLM | 3.6846 | 41.4586 |
|  |  |  | FASTmrMLM | 36.9348 | 6.8708 |
|  |  |  | ISIS EM-BLASSO | 3.224 | 0.0095 |
| HT | Marker668184 | 37 | mrMLM | 3.4772 | 35.9439 |
|  |  |  | ISIS EM-BLASSO | 8.0731 | 17.2554 |
|  | Marker479050 | 81 | FASTmrMLM | 3.5094 | 7.6881 |
|  |  |  | ISIS EM-BLASSO | 6.9027 | 28.9979 |
| DBH | Marker202441 | 225 | mrMLM | 4.1136 | 33.2391 |
|  |  |  | FASTmrMLM | 4.1833 | 16.6631 |
|  | Marker405019 | 161 | FASTmrMLM | 9.1754 | 25.7022 |
|  |  |  | FASTmrEMMA | 3.1949 | 9.9506 |
| RW | Marker439978 | 247 | mrMLM | 4.6444 | 17.1678 |
|  |  |  | FASTmrEMMA | 4.7221 | 17.1697 |
|  |  |  | ISIS EM-BLASSO | 4.9402 | 6.3555 |

**Supplementary Table S5**

**The information of the SNP located genes**

| **Trait** | **Marker** | **SNP Located gene** | **E-value** | **Gene function** |
| --- | --- | --- | --- | --- |
| HT | Marker463568 | RT_like super family | 8.65E-10 | Reverse transcriptase |
| RW | Marker248321 | RNase_H_like super family | 7.08E-13 | Endonuclease |
|  | Marker104674 | RNase_H_like super family | 4.44E-03 | Endonuclease |
|  | Marker342635 | RT_like super family | 1.52E-08 | Reverse transcriptase |
|  | Marker502947 | RT_like super family | 1.12E-08 | Reverse transcriptase |
|  | Marker513429 | RVT_2 super family | 1.12E-08 | Reverse transcriptase |
| VW | Marker103424 | RNase_H_like super family | 8.54E-09 | Endonuclease |
|  | Marker490183 | RT_like super family | 5.12E-06 | Reverse transcriptase |
| RYC | Marker151560 | FusA super family | 3.11E-03 | Translation elongation factor EF-G |
|  | Marker320005 | pepsin_retropepsin_like super family | 2.17E-03 | Cellular and retroviral pepsin-like aspartate proteases |
|  | Marker115068 | ribokinase_pfkB_like super family | 3.75E-10 | Kinases |
|  | Marker109152 | RNase_H_like super family | 1.11E-11 | Endonuclease |
|  | Marker104674 | RNase_H_like super family | 4.44E-03 | Endonuclease |
|  | Marker248321 | RNase_H_like super family | 7.08E-13 | Endonuclease |
|  | Marker216699 | RNase_H_like super family | 8.05E-05 | Endonuclease |
|  | Marker292651 | RNase_H_like super family | 2.17E-16 | Endonuclease |
|  | Marker174551 | RNase_H_like super family | 7.21E-06 | Endonuclease |
|  | Marker502947 | RT_like super family | 1.12E-08 | Reverse transcriptase |
|  | Marker238561 | RT_like super family | 3.23E-05 | Reverse transcriptase |
|  | Marker115413 | RT_like super family | 4.35E-08 | Reverse transcriptase |
|  | Marker405504 | RT_like super family | 1.88E-09 | Reverse transcriptase |
|  | Marker533819 | RT_like super family | 5.56E-12 | Reverse transcriptase |
|  | Marker169205 | RT_like super family | 8.68E-03 | Reverse transcriptase |
|  | Marker342635 | RT_like super family | 1.52E-08 | Reverse transcriptase |
|  | Marker144539 | rve super family | 8.17E-03 | Integrase core domain |
|  | Marker513429 | RVT_2 super family | 1.12E-08 | Reverse transcriptase |
|  | Marker375391 | RVT_2 super family | 3.74E-10 | Reverse transcriptase |
| RYC’ | Marker611740 | RNase_H_like super family | 4.87E-06 | Endonuclease |
|  | Marker482425 | RT_like super family | 3.74E-10 | Reverse transcriptase |
|  | Marker283415 | RT_like super family | 2.88E-11 | Reverse transcriptase |
|  | Marker248105 | RT_like super family | 1.79E-03 | Reverse transcriptase |

**Supplementary Table S6**

**Correlation analysis among masson pine traits**

| **Trait** | **HT** | **DBH** | **TW** | **VW** |
| --- | --- | --- | --- | --- |
| **DBH** | 0.507^**^ |  |  |  |
| **TW** | 0.256^**^ | 0.523^**^ |  |  |
| **VW** | 0.682^**^ | 0.958^**^ | 0.511^**^ |  |
| **RYC** | 0.331^**^ | 0.665^**^ | 0.969^**^ | 0.656^**^ |

Note: ^**^ meant significantly correlated at P<0.01
